# The Spelling Errors of French and English Children With Developmental Language Disorder at the End of Primary School

**DOI:** 10.3389/fpsyg.2020.01789

**Published:** 2020-07-21

**Authors:** Nelly Joye, Julie E. Dockrell, Chloë R. Marshall

**Affiliations:** Centre for Language, Literacy and Numeracy: Research and Practice, Department of Psychology and Human Development, UCL Institute of Education, University College London, London, United Kingdom

**Keywords:** spelling, cross-language, French, English, Developmental Language Disorder

## Abstract

Children with Developmental Language Disorder (DLD) often struggle learning to spell. However, it is still unclear where their spelling difficulties lie, and whether they reflect on-going difficulties with specific linguistic domains. It is also unclear whether the spelling profiles of these children vary in different orthographies. The present study compares the spelling profiles of monolingual children with DLD in France and England at the end of primary school. By contrasting these cohorts, we explored the linguistic constraints that affect spelling, beyond phono-graphemic transparency, in two opaque orthographies. Seventeen French and 17 English children with DLD were compared to typically developing children matched for age or spelling level. Participants wrote a 5 min sample of free writing and spelled 12 controlled dictated words. Spelling errors were analyzed to capture areas of difficulty in each language, in the phonological, morphological, orthographic and semantic domains. Overall, the nature of the errors produced by children with DLD is representative of their spelling level in both languages. However, areas of difficulty vary with the language and task, with more morphological errors in French than in English across both tasks and more orthographic errors in English than in French dictated words. The error types produced by children with DLD also differed in the two languages: segmentation and contraction errors were found in French, whilst morphological ending errors were found in English. It is hypothesized that these differences reflect the phonological salience of the units misspelled in both languages. The present study also provides a detailed breakdown of the spelling errors found in both languages for children with DLD and typical peers aged 5–11.

## Introduction

Language and literacy development are intricately related. Children build from their knowledge of sounds and words to progressively recognize and represent them in writing. On the one hand, awareness of speech sounds and the ability to manipulate them have been evidenced as an important predictor of later reading and spelling in a range of writing systems (Caravolas et al., 2012, 2013; Moll et al., 2014). On the other hand, it is also recognized that knowledge of word meaning supports the development of proficient reading (Nation and Snowling, 2004; Ricketts et al., 2007) and writing (Dockrell and Connelly, 2015).

It is thus unsurprising that many children with language difficulties also experience literacy difficulties. There is a well-documented comorbidity between Developmental Language Disorder (DLD) and dyslexia, with comorbidity rates ranging from 17 to 71% (Adlof and Hogan, 2018). In relation to spelling, a recent meta-analysis highlighted the spelling difficulties of children with DLD as compared to typical peers (Joye et al., 2019). The average adjusted standardized difference in spelling scores across studies was *g* = −1.42 (95% CI [−1.60; −1.24]) when children with DLD were compared to same-age typical peers, but non-significant when they were compared to younger children matched on language, reading or spelling, suggesting a clear spelling delay in this population. Furthermore, the meta-analysis highlighted that the difference in scores was particularly important in those children identified as having reading or phonological difficulties in addition to their language disorder.

At school entry DLD is reported to affect approximately 7.5% of the population (Tomblin et al., 1997; Norbury et al., 2016). The terminology and diagnostic criteria for language disorders have been the subject of debate in recent years (Ebbels, 2014). Lately, DLD has emerged as a preferred term from a consensus of experts (Bishop et al., 2016, 2017). DLD describes children who continue to experience language difficulties beyond the age of five, in the absence of any known medical condition, such as acquired brain injury or intellectual disability. DLD does not, however, exclude children with lower non-verbal ability scores (between −1 and −2 SD from the mean). It also recognizes that children with DLD may present with other developmental difficulties, especially dyslexia.

How language difficulties should be identified in pre-school (Dockrell and Marshall, 2015) and at school age (Bishop et al. 2016) is still a matter of debate. The taxonomy of linguistic difficulties experienced by children with DLD is broad (Rapin and Allen, 1987), and typically changes over time (Botting and Conti-Ramsden, 1999; Law et al., 2008), making identification a lengthy and unreliable process. Recently, the identification of language difficulties has increasingly turned to “markers” or “red flags” to pinpoint differences from typical language development (Visser-Bochane et al., 2017). Tasks such as sentence repetition and non-word repetition have been shown to be reliable indicators of language difficulties in a variety of languages at school age, alongside traditional measures of word and sentence production and comprehension (Conti-Ramsden et al., 2001; Leclercq et al., 2014). Amongst the potential clinical markers of language disorder identified in English, phonological and grammatical errors are recurrent in the literature. Specifically, omission of morphological inflections for the past tense (-*ed*), progressive (-*ing*) and noun plural/verb third person (-*s*), are commonly reported in the spontaneous language of English children with DLD (Leonard, 2014). These difficulties are, however, inconsistent, with children with DLD sometimes producing the target correctly, and sometimes not. Critically, differences in the rate of these grammatical errors are observed not only when children with DLD are compared to same-age children, but also when they are compared to younger children matched for language level (Leonard et al., 1992; Oetting and Horohov, 1997), suggesting that specific linguistic processes may be affected in DLD. Word-ending omissions are generally observed in preschool children with DLD, and become less apparent at school age (Bishop, 1994; Rice et al., 1998; Marchman et al., 1999).

The growing literature assessing clinical markers of DLD in languages other than English has challenged the universality of these specific phonological and grammatical errors as indicators of DLD in the early years (see Leonard and Kueser, 2019, for a discussion). For example, in French, clitic pronoun omissions have been proposed as potential markers of DLD (Leonard, 2016). French children with DLD produce an unusually high rate of object clitic errors in the early years (aged 3–7, Hamann et al., 2003) and at school age (5–13, Jakubowicz et al., 1998) and continue experiencing difficulties processing sentences with clitic pronoun cues even in late primary school (7–12, Maillart and Schelstraete, 2003). Difficulties with clitic pronouns are not the only markers of DLD in French. Consistent with English, difficulties have also been reported with verb morphology and in particular with the past tense (passé composé), which involves the auxiliary “être” (be) or “avoir” (have), often omitted (Jakubowicz, 2006). However, the data suggest that these difficulties may be restricted to the early years (Thordardottir and Namazi, 2007).

When it comes to potential school-age markers of DLD, spelling error analysis has provided useful insight into the continuing linguistic difficulties of children with DLD (Windsor et al., 2000; Bishop and Clarkson, 2003; Silliman et al., 2006; Larkin et al., 2013; Critten et al., 2014). In English, errors with verb endings (in particular past tense –*ed*) and noun plural –*s* are found in the spelling of children with DLD when compared to same-age peers, but results are inconsistent when comparisons are made with younger literacy- or language- matched controls (Silliman et al., 2006; Larkin et al., 2013; Critten et al., 2014). Furthermore, the ability of children with DLD to represent the *root* of derived and inflected words was found to be in line with their spelling level (Deacon et al., 2014), suggesting a subtle and specific difficulty with some morphological endings, rather than a broader morphological deficit. Spelling error analysis has also pointed to recurrent difficulties producing phonologically plausible spellings when children are compared to same-age peers, but not to younger peers matched for reading, language or spelling levels (Mackie and Dockrell, 2004; Larkin et al., 2013; Mackie et al., 2013; Critten et al., 2014). In French, spelling error analysis of samples of children with DLD in primary school has highlighted particular difficulties with word segmentation, and a high rate of phonologically implausible errors at the end of primary school in comparison to age-matched peers (Broc et al., 2013). To our knowledge, no spelling error comparison is available in French for children with DLD and younger ability-matched peers.

The comparison with spelling-matched peers is relevant to understanding the specific linguistic difficulties that might underlie spelling difficulties in children with DLD. If children with DLD present with specific phonological or morphological spelling errors over and above what might be expected given their spelling level, this would suggest that learning is specifically impaired in these areas. If, on the contrary, they present with error types commensurate with their spelling level, this would suggest an overall delay in all linguistic processes involved in spelling, commensurate with their language skills. This methodology has been used to characterize the spelling profiles of dyslexic children, pointing to subtle differences in their spelling, over and above those expected given their spelling level (e.g., specific difficulties with the silent letter *e*, Bourassa and Treiman, 2003; and with consonant clusters in English, Bruck and Treiman, 1990; or with long words in Danish, Juul and Petersen, 2017). If such differences could be found in children with DLD, they might be a marker of language difficulties in spelling, and a potential target for future interventions.

Together, the literature reviewed above suggests that children with DLD may have a long-lasting difficulty representing the sounds of words in spelling across languages, alongside more language-specific errors in primary school. However, to our knowledge, comparison with younger peers is rarely, if ever, provided in languages other than English, limiting the ability to draw conclusions on the specific linguistic mechanisms that may be affected in children with DLD, over and above their language and literacy levels (Joye et al., 2019). Another limitation in interpreting the current literature pertains to the inconsistency of tasks used to assess spelling. Whilst some studies have assessed children using a range of controlled words (Silliman et al., 2006; Broc et al., 2013; Critten et al., 2014) and pseudo-words (Larkin et al., 2013), others have used free writing tasks to assess spelling (Windsor et al., 2000; Mackie and Dockrell, 2004; Broc et al., 2013; Mackie et al., 2013), arguably giving children the advantage of choosing the words that they produce in the texts and thus allowing them to use words they are more confident in spelling. Furthermore, word and pseudoword lists have not consistently included morphologically- and orthographically- complex items (see for example McCarthy et al., 2012; Larkin et al., 2013), limiting investigations of spelling constraints in this population to the phono-graphemic conversion level.

French and English are two interesting orthographies to contrast for constraints in spelling development. Both are considered to be highly inconsistent in the phoneme-to-grapheme direction: approximately 20.9% consistency for French and 27.7% for English [although the grapheme-to-phoneme consistency is higher in French (87.6%) than in English (66.3%) – see estimation for monosyllabic words by Ziegler et al., 1996, 1997]. Both orthographies are phonomorphemic (i.e., they represent both sound and meaning units in spelling), and are governed by a range of orthographic rules and regularities (Pacton and Deacon, 2008). However, they differ on a number of other aspects critical to learning to spell beyond the early years: transparency and productivity of derivational morphology (Duncan et al., 2009; Casalis et al., 2015), transparency and richness of morphological inflections (McLeod, 2007), syllabic complexity (Seymour et al., 2003) and complexity and consistency of orthographic rules (Sprenger-Charolles, 2003). The sections below detail how the phono-graphemic, morphological and orthographic characteristics of French and English affect literacy development in these two languages, drawing specific hypotheses for the current study.

At the phono-graphemic level, inconsistencies do affect the rate and pattern of literacy development in French and English. Seymour et al. (2003) showed that children learning to read English took two years to reach about 70% of accuracy in familiar word reading, while French children reached this level after 1 year of being taught to read, and about 99% accuracy after 2 years. This was despite letter-sound correspondences being well-acquired by the end of the first year of instruction. In contrast, non-word reading reached only 64% accuracy in English, even after 2 years of reading instruction, against 97% in French. It is hypothesized that the relatively simple syllabic structure of French facilitates decoding, whilst its orthographic inconsistency makes mastering real word reading a slightly longer process. In English, both syllabic complexity and orthographic inconsistency affect the rate of learning to read (real and non-words) negatively. One particular area of difficulty in reading English pertains to the inconsistency of the vowel system (e.g., *beak, break and head*). In comparison, French vowel sounds are relatively consistent in the reading direction (- eau-, - au-, and -o- are always read /o/, Peereman and Content, 1997). When it comes to spelling real and non-words, English and French children also present with slightly different profiles. Caravolas et al. (2003) compared the spelling scores of poor and good spellers in third grade, on a parallel list of words and non-words matched for length and syllabic structure. Both French and English good spellers reached about 90% accuracy for words. However, differences were evident for non-words, where English good spellers spelled about 60% of the targets accurately, compared to 80% in French. Furthermore, phonological accuracy of the spelling attempts was poorer in the English than in the French sample. English spellers (good or poor) struggled with representing the syllabic structure of the words as compared to French spellers, and omitted unstressed vowels in particular. By contrast, French poor spellers were slightly more likely to omit consonants than vowels in their spelling. It is hypothesized that the syllable-timed stress pattern of French (Delattre and Olsen, 1969) has little effect on vowel production and thus facilitates the perception and written representation of these units, whereas the stress-timed pattern of English makes these units particularly difficult to perceive and spell. On the basis of the literature reviewed above, we thus expected errors at the phono-graphemic level on unstressed vowels in English in the present study, and on consonants in French. We further expected these errors to be particularly evident in the DLD groups, as this group typically experiences difficulties with phonological processing.

At the orthographic level, rules and regularities can also have an impact on spelling accuracy. For example, French children learn early on that, in order to spell the sound /g/ before the letters -*i*- and -*e*-, they have to add a -*u*- (e.g., *girafe* /ʒiʁaf/ -*giraffe*- and *genou* /ʒənu/ -knee- but *guitare* /gitaʁ/ -guitar- or *guêpe* /gɛp/ -wasp-), or that a “cédille” is needed in front of -*a*- or -*o*- for the letter -*c*- to make the /s/ sound (e.g., *cap* /kap/-cape- and *col* /kɔl/- collar-, but *glaçage*/glasaʒ/-icing- or *garçon* /gaʁsɔ̃/ -boy-). They also learn for example that the letter -*s*- needs to be doubled in order not to become sonorant between two vowels (e.g., *asile* /azil/-asylum- but *assis* /asi/-seated-). In English, orthographic constraints in phono-graphemic conventions also exist. For example, English children learn early on that long vowels are often spelled using the -*e*- letter at the end of CVC words (e.g., *pin* /pɪn/ but *pine* /pʌɪn/). Furthermore, in both languages, children have to choose between many alternatives in spelling vowels (e.g., /ɛ/ can be spelled *- in-, - ain-, - ein-, - im-, - aim-, -eim-*, /ɑ/ can be spelled - *en-, - an-, -em-*, or *-am-* and /bo/-*o-, -ô-, - au-, -eau-* in French, whilst the spelling for /i:/ has got as many as 13 grapheme representations in English: *mE, nicelY, thEmE, machInE, sEE, sEA, cAEsar, concEIve, nIEce, kEY, quAY, pEOple, and subpOEna*) (see Sprenger-Charolles and Béchennec, 2004, for a comprehensive review of orthographic constraints in learning to read and spell French and English). Because of the complex nature of orthographic constraints in both languages, we expected young and DLD spellers in our study to have difficulties with these rules. Specifically, we expected long and complex vowel spelling and phoneme-grapheme correspondences dependent on orthographic rules to be difficult for these groups in both languages. At the morphological level, little is known about the differential role of derivational morphology in literacy development in French and English. To our knowledge, only two studies have assessed this aspect of morphology comparatively in these two languages. One examined the ability to derive words and pseudowords orally in grade 1–3 French and English children (Duncan et al., 2009). The other assessed word decoding in a set of words and pseudowords that were or were not derived, in a population of grade 4 French and English children (Casalis et al., 2015). Taken together, their results suggest that French children have an earlier and more proficient awareness of derivation processes in word formation than their English peers. They were more likely to successfully use this process to produce derived words and pseudowords orally and judge their acceptability in grades 1–3 (Duncan et al., 2009) or to decode them in grade 4 (Casalis et al., 2015). On the basis of these results, we expected our French sample to perform well in spelling derived words compared to their English peers, and we included specifically derived items in our word lists to assess this aspect of morphological spelling.

Finally, inflectional morphology differs greatly in French and English spelling. The range of morphological markers is much greater in French than in English. Nouns are inflected not only for number (final -*s*, exceptionally -*x*), but also for gender (feminine -*e*). Verbs are inflected for all tenses and persons in French (as opposed to just the third person, past tense and present progressive in English). As an example, the French present for verbs ending in *–er* (e.g., *chant****er*,** *to sing)* has no less than five different inflections (-*e, -es, -ons, -ez*, and -*ent*): *Je chant****e*** *(I sing), tu chant****es*** *(you sing), il chant****e*** *(he sing****s****), nous chant****ons*** *(we sing), vous chant****ez*** *(you (pl.) sing), ils chant****ent*** *(they sing)*. This is known to be a challenge to French spelling and there is a strong emphasis on grammatical spelling early on in French education (Morin et al., 2018). By contrast inflectional morphology in English is comparatively simple. There is no gender marking in the noun phrase, only the plural, marked by a regular -*s* ending, (which is heard as /z/, /s/ or /ɪz/ depending on the phonological context) and possessive marking (using the apostrophe *–‘s* or –*s’* and realized phonologically like a plural). In a few irregular cases plural may provoke a phonological change in the stem as in *foot*/*feet*, *woman*/*women, scarf/scarves* or *stimulus/stimuli*. The past tense for verbs is marked by -*ed* (heard as /t/, /d/, or /ɪd/ depending on the context), except for a set of irregular verbs, which also see their stem altered (e.g., *buy*/*bought*, *stand*/*stood*). The present progressive is marked by -*ing* and the third person present by -*s*. Inflectional morphology in English is introduced early in the school curriculum, and largely mastered within the first year of schooling. For example, the plural -*s* is mastered as early as the first semester of grade 1 in English-speaking children (Treiman, 1993; Turnbull et al., 2011). Furthermore, and critically for the population of children with DLD, morphological inflections are typically heard in English (-*s*, -*ing*, and -*ed* are pronounced in oral language as well as represented in spelling), whereas there are many silent and homophone inflections in French (e.g., the plural *-s*, or feminine *-e* are often silent in French, and the verb endings *-er*, *-é*, *-ait*, *-ais* or *-aient* all sound the same, see the review by Pacton and Deacon (2008), on French and English morphology). In the present study, by comparing English children with DLD, who may be able to spell word endings either by ear or by application of their morphological knowledge, to a group of French children, who necessarily need to apply morphological knowledge in their spelling of word endings, the present study aims to shed further light on the mechanisms underlying these specific error types. If found in both populations of children with DLD, as compared to spelling-matched peers, morphological ending omissions might be indicative of a specific underlying morphological deficit. If found only in the English-speaking population of children with DLD, they might instead reflect underlying phonological difficulties. It is indeed still a matter of debate whether the word ending omissions produced by children with DLD are phonological or grammatical in nature. Final *-s* and *-ed* are relatively non-salient units to perceive in oral language, and it has been hypothesized that deficits in the phonological representation of children with DLD might impair their production of these discrete units (Marshall and van der Lely, 2007; Parisse and Maillart, 2007).

Given the limited research into spelling development across languages beyond the early years and the lack of characterization of spelling profiles in children with DLD at school-age, the present study’s aims were twofold:

• To investigate French-English cross-language differences in spelling development beyond the first 2 years of literacy instruction.
• To provide a detailed profile of the specific spelling errors produced by children with DLD learning to spell in French or English at the end of primary school, as compared to both age- and spelling-matched typical peers.

## Materials and Methods

### Participants

Participant recruitment and data collection procedures were approved by our institution’s ethics committee and in line with current data management regulation. All children and their parents/guardians gave their informed consent before participation. One hundred and two participants were recruited from five schools in the South-East of England and seven schools in the South-East and North of France. The same recruitment process was used in both countries. Mainstream schools with a language unit were approached, as well as mainstream schools with a known caseload of children with DLD. Language units (“ULIS-école” in France) are specialist units within mainstream schools, where children with language disorders receive specialist instruction for some of the curriculum and are included in the mainstream classroom for the rest of their learning. Teachers, speech and language therapists and Special Educational Needs Coordinators (SENCOs, in the United Kingdom) were consulted verbally, and parents were consulted using a brief questionnaire (within the consent form), in order to identify children experiencing language and literacy difficulties within the language units and mainstream Year 3, 4, 5, and 6 classes (ages 8–11).

Children were further tested to ascertain their language difficulties using standardized measures. Three measures were taken: sentence repetition, word comprehension and sentence comprehension. Children’s diagnosis of DLD was confirmed and they were included in the DLD sample if they scored −1.28 SD or below in at least two of these measures, or on a composite of all three measures. Children reaching scores −2 SD or below on a standardized test of Non-Verbal Performance (NVP) were excluded. Following this procedure, 17 children with language difficulties were identified within the French sample and 17 in the English sample.

A further 17 typically developing children matched on chronological age (CA), and 17 younger children (SA) matched on the raw spelling scores of the Wechsler Individual Achievement Test (WIAT), were identified in each country. These children had NVP, language and spelling scores within the norm for their age range, as reported by parents and teachers and measured on standardized tasks. Table 1 provides a summary of the groups’ characteristics.

**TABLE 1:** Characteristics of the participants.

	FR			EN		
	CA	DLD	SA	CA	DLD	SA
N	17	17	17	17	17	17
N boys	6	13	8	7	10	8
N children with other known diagnoses (ADHD and/or dyslexia)	0	7	0	0	7	0
N children scoring below −1 SD on the word reading measure	0	15	5	0	11	1
N children scoring below −1 SD on the phonological awareness measure	0	5	2	0	7	3
N children who speak other languages at home	1	2	1	0	4	0
Age	10.15 (0.73)	10.14 (0.83)	7.85 (1.04)	9.82 (0.7)	9.94 (0.99)	6.89 (0.86)
Language Composite	−0.12 (0.56)	−2.3 (1.08)	0.09 (0.65)	−0.1 (0.73)	−1.86 (0.45)	−0.07 (0.49)
Non-verbal performance	0.41 (0.74)	−0.2 (0.89)	0.15 (1.05)	0.55 (0.71)	−0.47 (1)	0.27 (0.97)
Word Reading Accuracy	0.36 (0.41)	−2.79 (1.53)	−0.67 (0.93)	0.71 (0.9)	−1.25 (0.9)	0.59 (1.07)
Phonological awareness	0.75 (0.4)	−0.75 (1.25)	0.13 (0.84)	0.57 (0.36)	−0.57 (1.06)	0 (0.94)
WIAT spelling	−0.07 (0.66)	−1.83 (0.75)	0.21 (0.75)	0.69 (0.72)	−1.45 (0.48)	0.34 (1.1)
WIAT spelling raw score	31.18 (4.14)	17.35 (6.77)	19.76 (5.01)	36.24 (6.25)	22 (3.18)	21.29 (3.2)

*Age is expressed in years, and all other scores are *Z*-scores, calculated from the mean and standard deviations of the test’s results for the child’s age group when available, or from the sample’s mean and standard deviation stratified by country and age group.*

As indicated in Table 1, children in France (FR) were marginally older than their English peers (EN). This is because French children start formal literacy instruction at age 6, whilst English children start at age 5. Years of instruction were preferred over developmental age for matching, as we considered spelling to be a skill highly dependent on explicit instruction in school. The French and the English TD samples were representative of the general population, as evidenced by their spelling, reading, NVP and language composite standard scores.

### Measures

#### Control Measures

Standardized assessments of language, word reading, phonological awareness and NVP were administered as grouping and control measures. Table 2 provides a description of the parallel tasks used to assess language, reading, phonological awareness and NVP in both languages.

**TABLE 2:** Characteristics of the parallel French and English grouping and control measures.

Ability	French test	Task	Rel./Val.	English test	Task	Rel./Val.
Language – Sentence repetition	L2MA2 – Répétition de phrases	Child repeats 15 sentences of increasing complexity	NA/0.85**	CELF-4 – Recalling sentences	Child repeats 32 sentences of increasing length	0.91/0.84**
Language – Sentence comprehension	BALE – Oral language	Child chooses 1 out of 4 pictures that goes with the sentence given. 20 items. No discontinuation rule.	NA/NA	TROG-2	Child chooses 1 out of 4 pictures that goes with the sentence given. Up to 20 blocks of 4 items. Discontinued after 5 blocks failed.	0.87/0.54*_a_
Language - Word Comprehension	BALE – Oral language	Child chooses 1 out of 6 pictures that goes with the word given. 15 items. No discontinuation rule.	NA/NA	BPVS-3	Child chooses 1 out of 4 pictures that goes with the word given. Up to 14 blocks of 12 items. Discontinued after 8 or more errors within a block	NA/NA
Non-verbal Performance – Matrices	Raven’s Colored Progressive Matrices	Child chooses 1 out of 6 figures to fill in a pattern. 3 sets of 12 patterns to complete. No discontinuation rule.	0.80/0.91*_b_	Raven’s Colored Progressive Matrices	Child chooses 1 out 6 figures to fill in a pattern. Three sets of 12 patterns to complete. No discontinuation rule.	0.80/0.91*_b_
Reading – Timed word reading	BALE – Regular and irregular word reading (low frequency)	Child reads 20 regular words and 20 irregular words. No discontinuation rule.	NA/NA	BAS-3 – Word reading list A	Child reads up to 90 words of increasing complexity (discontinued after 8 failures within a block of 10)	0.98/0.89*_c_
Phonological Awareness – syllable, rime and phoneme extraction	Parallel bespoke task	Child extracts the common unit in 24 pairs of disyllabic words (8 syllable, 8 rime and 8 phoneme pairs).	NA/NA	Parallel bespoke task	Child extracts the common unit in 24 pairs of disyllabic words (8 syllable, 8 rime and 8 phoneme pairs).	NA/NA
Spelling – Word spelling	WIAT-CDN-FR – Spelling	Child spells up to 53 words of increasing complexity (discontinued after 6 misspellings)	0.91/NA	WIAT-UK-II – Spelling	Child spells up to 53 words of increasing complexity (discontinued after 6 misspellings)	0.94/78*_d_

CELF-4: Clinical Evaluations of Language Fundamentals, 4th edition UK (Semel et al., 2006), L2MA2: “Langage oral, Langage écrit, Memoire, Attention,” 2nd edition (Chevrie-Muller et al., 2010), TROG-2 : Test of Reception of Grammar, 2nd edition (Bishop, 2003), BALE: “Batterie Analytique du Langage Ecrit” (Jacquier-Roux et al., 2010), BAS-3: British Ability Scale, 3rd edition (Elliott and Smith, 2012), Parallel bespoke French-English phonological awareness task adapted from Duncan et al. (2006), Raven’s matrices (Raven et al., 1998a, b), WIAT-UK-II: Wechsler Individual Achievement Test – 2nd UK Edition (Wechsler, 2005b), WIAT-CDN-FR: Wechsler Individual Achievement Test, Canadian Edition (Wechsler, 2005a), Rel. Reliability, Val. Validity; *concurrent validity, **construct validity, _a_ with ‘concepts and following directions’ from the CELF-3 _b_ with WISC-R, _c_ with list B of the same test, _d_ with WRAT3, NA, not available.

#### Personal Narrative Text

**FIGURE 1:**
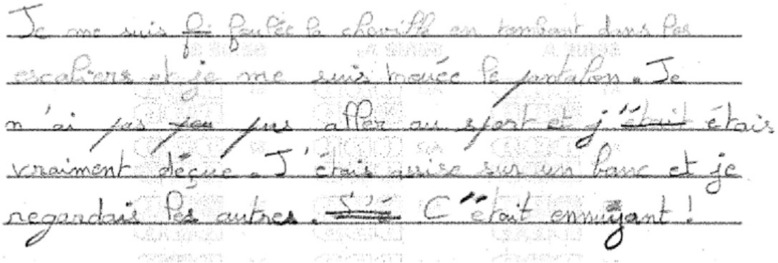
Example of a text produced by a French child aged 10 years 11 months (year 5).

**FIGURE 2:**
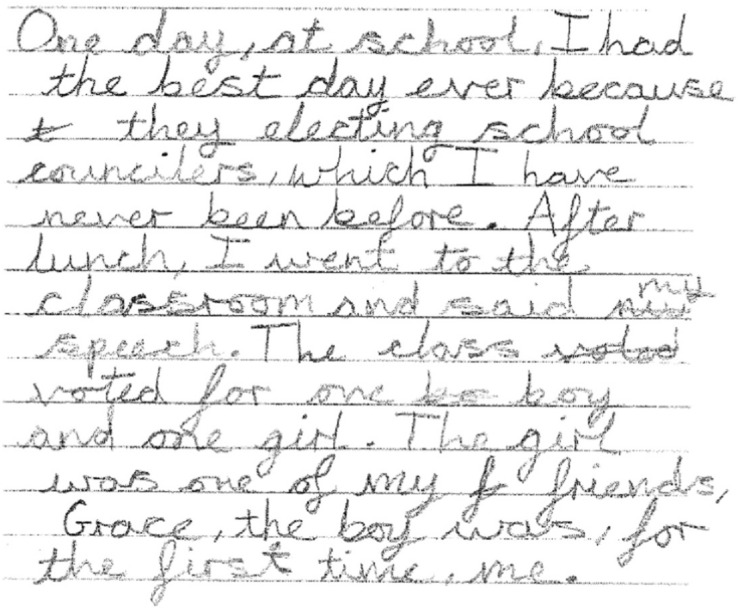
Example of a text produced by an English child aged 10 years 11 months (year 6).

A naturalistic sample of writing was obtained using a narrative task. The narrative Curriculum-Based Measure for Writing (CBM-W) from Dockrell et al. (2014) was used in both languages. The task was administered in small groups. Children chose to write to one of the following prompts: “One day, I had the best day ever…” (“Un jour, j’ai eu la meilleure des journées…”) or “One day, I had the worst day ever…” (“Un jour, j’ai eu la pire des journées…”). Children were given a lined A4 sheet with the prompt, and told they were to write the best story possible within 5 min. They were given 30 s to think about their story before they started writing. At the end of the 5 min, children finished their last sentence and put their pens down. The proportion of words spelled correctly in this task has good construct validity (0.87) as an accuracy measure and a 0.30 consistency level with the WOLD-writing overall expression score (Dockrell et al., 2014). Figures 1, 2 provide an example of production from a TD French and an English participant respectively.

#### Twelve Dictated Spelling Words

The 12 dictated words were chosen for analysis from the French and English version of the WIAT-spelling test (Wechsler, 2005a, b). All children were administered the full test as a group, in order to obtain their standard score. Each word was given verbally to children, then used in its sentence context, and then given again in isolation for children to spell, as per the test manual’s instructions. A subset of the words commonly misspelled by children in both languages was then selected for further analysis. Words were chosen to be representative of the phonological, orthographic, morphological and semantic conventions of written French and English. Words were also matched across languages on number of letters, phonemes, and as much as possible on frequency counts. Accuracy scores on the 12 words were highly correlated with raw scores on the full WIAT scale, both in French (*r* = 0.94) and in English (*r* = 0.96). Cronbach’s Alpha was 0.85 on the French scale, and 0.89 on the English scale, indicating good reliability. Table 3 provides a list of the 12 words chosen in each language.

**TABLE 3:** Characteristics of the words chosen from the parallel WIAT-spelling tasks.

FR	Freq	NbPhon	NbLet	EN	Freq	NbPhon	NbLet
main	684	2	4	big	2666	3	3
gros	757	3	4	hand	295	4	4
plomb	19	3	5	careless	3	5	8
sautait	7	4	7	strength	22	6	8
grimpa	1	5	6	riding	143	5	6
plafond	29	5	7	climbed	373	5	7
suis	855	3	4	guess	127	3	5
excitation	3	9	10	right	852	3	5
mer	521	3	3	knew	270	3	4
dois	117	3	4	patients	38	6	8
soupçon	3	5	7	ceiling	35	5	7
aujourd’hui	249	7	11	couldn’t	NA	5	8
Mean (*SD*)	271.54 (335.15)	4.33 (2.02)	6 (2.52)	Mean (*SD*)	438.55 (778.25)	4.42 (1.16)	6.08 (1.83)

*EN, English word targets; Freq, English: Frequency per million (=number of occurrences of target word/number of all word occurrences in database × 1,000,000), French: Estimated frequency of Usage per million (U = number of occurrences of target word/number of all word occurrences in database × dispersion of the frequencies across readers × 1,000,000); NbPhon, number of phonemes; NbLet, number of letters; FR, French word targets. The indices given above were all taken from the Children’s word printed database for English (Masterson et al., 2010) and from the Manulex database for French (Lété et al., 2004).*

### Procedure

Both experimental tasks were administered in small groups of up to eight children, in one 30-min session. The non-verbal performance test was also administered during this first session, following the test manual’s instructions for group administration Children were then seen individually to assess their language, phonological awareness and reading skills.

#### Productivity and Accuracy Measures for the Text and Dictated Words

For the texts, productivity was measured in number of words produced by each child, excluding proper nouns and illegible words. For both tasks, accuracy was measured by dividing the number of words correctly spelled by the number of words attempted.

#### Qualitative Analysis of Spelling Errors

After measuring accuracy in the texts and dictated words, a qualitative coding of the spelling errors was conducted by the first author and by two trained independent raters who were native speakers of each language. The framework for spelling error analysis was adapted from Apel and Masterson (2001). Spelling errors were classified as either phonological, orthographic, morphological or semantic in nature, as detailed in Table 4. Subcategories were attributed to specific error types within these broad categories, for a fine-grained characterization of spelling profiles in children with DLD, as shown in Table 4. Cohen’s Kappa between raters was 0.82 (88% agreement) in English and 0.76 (81% agreement) in French. Rate of errors in each category is given in number of errors per word produced, in order to account for individual differences in productivity.^1^

**TABLE 4:** Coding of spelling errors, adapted from Apel and Masterson (2001).

Overall category	Fine-grained coding	Definition	Example (FR)	Target (FR)	Example (EN)	Target (EN)
PHON – Errors where the child did	PHON-OM-vow	Omission of a stressed vowel	*frpé	frappé	*destintion	destination
not represent the phonological skeleton of the word	PHON-OM-cons	Omission of an obligatory consonant	*tabeau	tableau	*chool	school
	PHON-SUB-vow	Substitution of a stressed vowel	*lou	les	*dack	duck
	PHON-SUB-cons	Substitution of a consonant	*pardi	parti	*den	then
	PHON-ADD	Addition of a phoneme	*lavai	avait	*minunts	minutes
ORTH - Errors where the child did not call on relevant orthographic	ORTH-IRR-silent	Omission of an unpredictable silent letter	*plafon	plafond	*climed	climbed
knowledge in his/her production	ORTH-IRR-cons	Substitution of an ambiguous consonant spelling	*cand	quand	*squeesing	squeezing
	ORTH-IRR-vow	Substitution of an inconsistent long vowel grapheme	*ancre *copin	encre copain	*laiter *hed	later head
	ORTH-IRR-vow	Substitution or omission of an unstressed vowel grapheme	N/A	N/A	*apon *favrite	upon favourite
	ORTH-IRR-accent	Error on an accent	*embéter	embêter	N/A	N/A
	ORTH-IRR-MGR	Error of letter inversion	*avce	avec	*beacuse	because
	ORTH-REG	Error on a regular spelling pattern	*poto	poteau	*sista	sister
	ORTH-RUL	Error on a taught spelling rule or an illegal letter sequence	*grinpa	grimpa	*recieve *annd	receive and
	ORTH-PHON	Error with orthographically constrained graphemes-phoneme correspondences affecting phonology	*amourese *gour	amoureuse jour	*tims *techer	times teacher
	ORTH-MOR	Error with rule-constrained applications of inflections and derivations	*obligait	obligeait	*realy *blammed	really blamed
MOR – Errors where the child did	MOR-INF-gender	Error on gender inflection	rempli	remplie	N/A	N/A
not call on relevant morphological	MOR-INF-tense	Error on tense inflection	demander	demandé	*happend	happened
knowledge in his/her production	MOR-INF- Person	Error on person marking	avais	avait	*comse	comes
	MOR-INF-Number	Error on number marking	copain	copains	way’s	ways
	MOR-INF-Poss	Error on possessive marking	N/A	N/A	teachers	teacher’s
	MOR-DER-base	Error on the base of a complex word	*gran	grand	ment	meant
	MOR-DER-Pre	Error on the prefix of a complex word	*extrordinaire	extraordinaire	*extrordinary	extraordinary
	MOR-DER-Suff	Error on the suffix of a complex word	*maîtrèsse	maîtresse	assemble	assembly
	MOR-CON	Errors on word contractions	*quon	qu’on	*I’am	I’m
	MOR-PHON	Omission of a morphological marker affecting phonology	grand le	grande les	head (verb) goal	headed goals
SEM - Errors on the meaning of the word attempted	SEM-SEG	Segmentation errors	*les cole *on n’a	l’école on a	*some thing	something
	SEM-HOMO	Homophone errors (within the same grammatical category)	poing	point	peace	piece
	SEM-MOR	Use of a grammatical homophone	et à	est a	their your	there you’re
	SEM-PHON	Wrong word choice: use of another word, affecting semantics and phonology	j’ai	j’aime	were	wear

## Results

Productivity and accuracy results are presented first, followed by the qualitative analysis of the spelling errors. These results are always presented for the language comparison first (French vs. English), and then for the subgroup comparisons (CA vs. DLD vs. SA) within each language. Finally, regression models to predict a subset of outcome spelling measures are presented.

### Productivity and Accuracy Within and Across Languages

Robust ANOVAs and *post hoc* tests were run to assess language and subgroup effects on productivity and accuracy measures, in order to account for the presence of outliers and the heterogeneity of variance (Mair and Wilcox, 2020). A robust measure of effect size (*ξ*) was computed where relevant. *ξ*-values of 0.10, 0.30, and 0.50 correspond to small, medium, and large effect sizes respectively (Wilcox and Tian, 2011). Table 5 presents the mean and standard deviation for the productivity and accuracy measures in the two tasks for our groups of interest.

**TABLE 5:** Mean and standard deviation for the spelling productivity and accuracy measures.

		FR			EN		
		CA	DLD	SA	CA	DLD	SA
Written texts	Words attempted	35 (5)	15 (3)	20 (3)	51 (4)	25 (6)	21 (5)
	Proportion of words correct	0.75 (0.03)	0.45 (0.07)	0.44 (0.09)	0.95 (0.02)	0.81 (0.02)	0.74 (0.03)
12 words	Words attempted	12 (0)	12 (0)	12 (0)	12 (0)	12(0)	12 (0)
	Proportion of words correct	0.62 (0.05)	0.22 (0.07)	0.24 (0.07)	0.85 (0.03)	0.30 (0.04)	0.29 (0.02)

#### Language Comparison

In the texts, English children produced more words (*F*(1) = 6.70, *p* = 0.013, *ξ* [95% CI] = 0.32 [0.07-0.52]) than French children. On average, English texts were 10 words longer than the French texts. There was no difference in productivity in the word dictation task, with all children attempting all 12 words dictated.

English children produced a higher rate of correct words in the texts (*F*(1) = 50.15, *p* < 0.001, *ξ* [95% CI] = 0.79) than French children. On average, English children produced a misspelling every six words of their texts, whilst the French children produced a misspelling every second word. Similarly, in dictation, word accuracy was higher in English than in French (*F*(1) = 10.03, *p* = 0.003, *ξ* [95% CI] = 0.08 [0–0.39]). On average, there was a misspelling in every word attempted in both the English and the French dictated words.

#### Subgroup Comparisons

In both languages, children with DLD and SA peers produced shorter (*F*(2) = 39.00, *p* = 0.001, *ξ* [95% CI] = *0*.71 [0.46–0.86]) and less accurate (*F*(2) = 53.21, *p* = 0.001, *ξ* [95% CI] = 0.65 [0.39–0.89]) texts than their CA peers. On average, children with DLD and SA peers produced a misspelling every two/three words, whilst CA peers produced a misspelling every seven/eight words in their texts. In word dictation, word accuracy was better in the CA than in the DLD and SA groups (*F*(2) = 169.92, *p* < 0.001, *ξ* [95% CI] = 0.99 [0.76–0.99]), in both languages. On average, there was a misspelling in every word attempted in the DLD and SA samples, and one misspelling every three to four words in the CA samples.

### Qualitative Analysis of Spelling Errors

**FIGURE 3:**
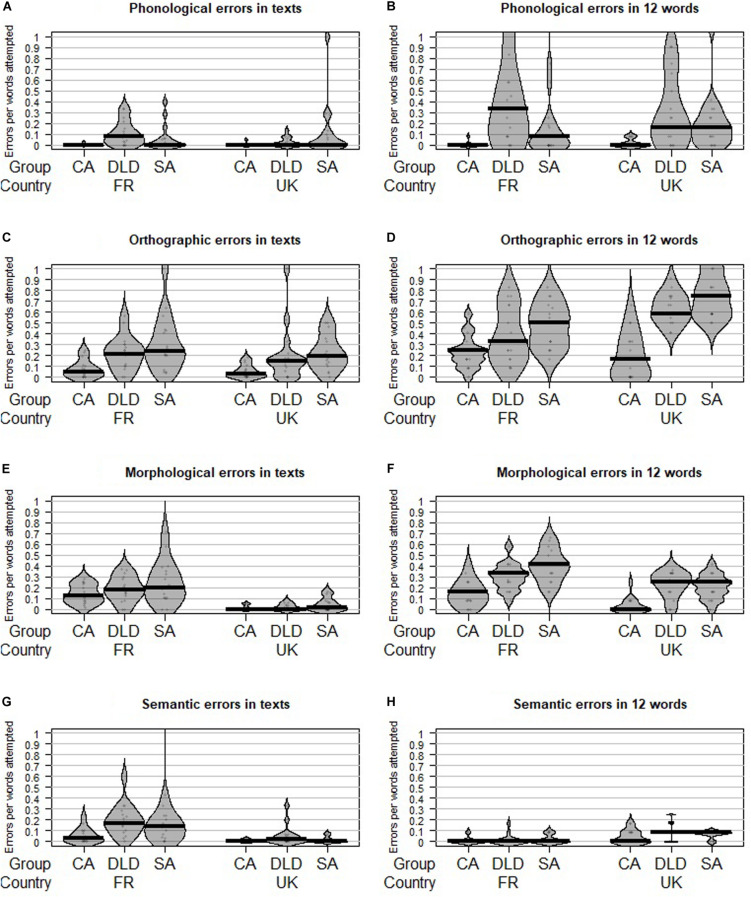
Median and distribution of the proportion of each error type, per language and group.

Results from the qualitative error coding were analyzed using Wilcoxon rank-sum tests, to account for the overall positive skewness and heterogeneity of variance in the data. A Bonferroni correction for multiple comparisons was applied to reduce the chance of false positives. *P*-values below 0.005 were considered significant. Figure 3 presents bean plots for the proportion of each error type, per language and group, in the texts and dictated words. The bean plots represent the median, data points and a bean-shape smoothed density curve (verticalized), showing the non-normal distribution of the data across all error types. Results from the Wilcoxon rank-sun tests are given in turn for the language comparisons in both tasks, and for the subgroup comparisons, within each language and for both tasks. Error types are then further broken down using the fine-grain coding scheme, for each type of error (phonological, orthographic, morphological, and semantic), in order to provide a detailed profile of the types of errors made within each language and group.

#### Phonological Errors

As shown in Figures 3A,B, the rate of phonological errors was not significantly different across languages in the texts (*W* = 1486.5, *p* = 0.16, *r* = 0.14) or dictated words (*W* = 1228, *p* = 0.61, *r* = 0.05).

In the French texts, children with DLD produced a higher rate of phonological errors than their CA peers (*W* = 245, *p* < 0.001, *r* = 0.64). This was the only significant difference (CAvsSA: *W* = 103, *p* = 0.08, *r* = 0.30; DLDvsSA: *W* = 199, *p* = 0.05, *r* = 0.33). The same result was found in the French dictated words, with more errors in the DLD than in the CA samples (DLDvsCA: *W* = 257.5, *p* < 0.001, *r* = 0.72; CAvsSA: *W* = 79, *p* = 0.006, *r* = 0.46; DLDvsSA: *W* = 202, *p* = 0.05, *r* = 0.34).

In the English texts, the rate of phonological errors did not significantly differentiate any of the subgroups (DLDvsCA: *W* = 191, *p* = 0.04, *r* = 0.35, CAvsSA: *W* = 122.5, *p* = 0.41, *r* = 0.14, DLDvsSA: *W* = 86.5, *p* = 0.01, *r* = 0.42). However, in the dictated words, English children with DLD (*W* = 243, *p* < 0.001, *r* = 0.62) and SA peers (*W* = 48, *p* < 0.001, *r* = 0.61) produced a higher rate of phonological errors than their CA peers, with no other group differences (DLDvsSA: *W* = 164, *p* = 0.51, *r* = 0.11).

In both languages and tasks, phonological errors consisted largely of consonant omissions (especially in consonant clusters, e.g., *ecept* for *except*) and vowel (e.g., *dack* for *duck*) and consonant substitutions (e.g., *den* for *then*). See Appendices A, B for the breakdown of error types within the phonological category.

#### Orthographic Errors

As shown in Figures 3C,D, the rate of orthographic errors was not significantly different across languages in the texts (*W* = 1547.5, *p* = 0.10, *r* = 0.16). However, in the dictated words, the difference in rates of orthographic errors between English and French children approached significance (*W* = 885.5, *p* = 0.0054, *r* = 0.28), with more orthographic errors in the English word samples.

In the French texts, children with DLD (*W* = 229, *p* = 0.004, *r* = 0.50) and SA peers (*W* = 48, *p* < 0.001, *r* = 0.57) produced a higher rate of orthographic errors than CA peers, but did not differ significantly from one another (DLDvsSA: *W* = 125, *p* = 0.51, *r* = 0.11). By contrast, in the dictated words, only SA peers produced a higher rate of orthographic errors than CA peers (*W* = 48, *p* < 0.001, *r* = 0.57), with no other group differences (DLDvsCA: *W* = 188.5, *p* = 0.13, *r* = 0.26; DLDvsSA: *W* = 108.5, *p* = 0.22, *r* = 0.21).

In the English texts, SA children produced a higher rate of orthographic errors than CA peers (*W* = 40.5, *p* < 0.001, *r* = 61), all other group comparisons being non-significant (DLDvsCA: *W* = 206.5, *p* = 0.03, *r* = 0.36; DLDvsSA: *W* = 108.5, *p* = 0.22, *r* = 0.21). A slightly different trend was observed in the dictated words, where English children with DLD (*W* = 273, *p* < 0.001, *r* = 0.76) and SA peers (*W* = 9, *p* < 0.001, *r* = 0.80) both performed worse than their CA peers.

In the French texts and dictated words, orthographic errors were largely found on irregular vowel (e.g., *rancontre* for *rencontre*) and consonant spellings (e.g., *commense* for *commence*), regular orthographic patterns (e.g., *poto* for *poteau*) and silent letters (e.g., *pui* for *puis*). In the English texts, errors on unstressed (e.g., *choclate* for *chocolate*) and long vowels (e.g., *laiter* for *later*), regular orthographic patterns (e.g., *netle* for *nettle*) and contextual spelling dominated (e.g., *gat* for *gate*), especially in the younger and DLD groups. In the dictated words, English children also produced orthographic errors on silent letters (e.g., *climed* for *climbed*) and irregular vowel and consonant spellings (e.g., *sealing* for *ceiling*). See Appendices C, D for the breakdown of error types within the orthographic category.

#### Morphological Errors

As shown in Figures 3E,F, French children produced a higher rate of morphological errors than their English peers in their texts overall (*W* = 2341.5, *p* < 0.001, *r* = 0.70). The same result was observed in the dictated words (*W* = 1884, *p* < 0.001, *r* = 0.39).

In the French texts, the rate of morphological errors was not significantly different across any of the groups (DLDvsCA: *W* = 189, *p* = 0.13, *r* = 0.26; DLDvsSA: *W* = 127, *p* = 0.56, *r* = 0.10; SAvsCA: *W* = 94.5, *p* = 0.09, *r* = 0.29). However, in the dictated words, French children with DLD (*W* = 232, *p* = 0.002, *r* = 0.52) and SA peers (*W* = 29, *p* < 0.001, *r* = 0.34) produced a higher rate of morphological errors than their CA peers.

The pattern was similar in English, with no significant difference in the rate of morphological errors in the texts across groups (DLDvsCA: *W* = 171, *p* = 0.30, *r* = 0.18, DLDvsSA: *W* = 121, *p* = 0.41, *r* = 14, SAvsCA: *W* = 99, *p* = 0.08, *r* = 0.30), but differentiated results in the dictated words. In the dictated words, English children with DLD (*W* = 259, *p* < 0.001, *r* = 0.70) and SA peers (*W* = 26.5, *p* < 0.001, *r* = 0.72) also produced a higher rate of morphological errors than their CA peers.

In the French texts, children produced a large number of morphological errors on tense (e.g., *aller* for *allé*), number (e.g., *les table* for *les tables*) and person inflections (e.g., *j’était* for *j’étais*). In the English DLD and SA samples, the majority of morphological errors were omissions of inflections (e.g., *tell* for *tells*). The pattern was slightly different in the dictated words, where French children also produced many derivational base errors (e.g., *sotait* for *sautait*). In the English dictated words, in addition to inflection omissions, children with DLD and SA also produced errors with contractions (e.g., *could’nt* for *couldn’t*), derivational suffixes (e.g., *strengcth* for *strength*) and tense marking (e.g., *climbd* for *climbed*). See Appendices E, F for the breakdown of error types within the morphological category.

#### Semantic Errors

As shown in Figures 3G,H, French children produced a higher rate of semantic errors than English children in their texts overall (*W* = 2118.5, *p* < 0.001, *r* = 0.56). The trend was reversed in the dictated words (*W* = 629.5, *p* < 0.001, *r* = 0.51), where English children produced more semantic errors than their French peers. Note, however, that this error type was marginal in both languages in the dictated words, with error rates flooring close to zero per word attempted.

In the French texts, children with DLD produced a higher rate of semantic errors than CA peers (*W* = 236, *p* = 0.002, *r* = 0.54) but there were no other group differences (DLDvsSA: *W* = 168.5, *p* = 0.42, *r* = 0.14; SAvsCA: *W* = 82.5, *p* = 0.03, *r* = 0.36). However, in the dictated words, no group difference appeared, with the error rate being close to zero across groups (DLDvsCA: *W* = 145, *p* = 1, *r* = 0; CAvsSA: *W* = 128.5, *p* = 0.42, *r* = 0.14; DLDvsSA: *W* = 129, *p* = 0.44, *r* = 0.13).

The rate of semantic errors was close to zero in the English texts and did not differentiate any of the groups (DLDvsCA: *W* = 192, *p* = 0.07, *r* = 0.31; DLDvsSA: *W* = 181, *p* = 0.16, *r* = 0.24; SAvsCA: *W* = 140, *p* = 0.87, *r* = 0.03). The same was observed in the dictated words (DLDvsCA: *W* = 209.5, *p* = 0.02, *r* = 0.41; CAvsSA: *W* = 99, *p* = 0.08, *r* = 0.30; DLDvsSA: *W* = 179.5, *p* = 0.15, *r* = 25).

In the French texts, children with DLD and SA produced a large proportion of segmentation errors (*je ma muse* for *je m’amusais*). In all groups, errors on grammatical homophones were also prominent (e.g., *à* for *a* or vice versa). These errors were almost absent in English texts. In the dictated words, however, English children, especially in the DLD and SA subgroups produced some errors with grammatical homophones (e.g., *new* for *knew*) and word choice (e.g., *guest* for *guess*). See Appendices G, H for the breakdown of error types within the semantic category.

### Controlling for Sampling Differences Across Languages

In order to control for any sampling confounds that could explain the cross-language differences observed, we ran further regression analyses. These regressions examined the following predictors: age, NVP and phonological awareness as control variables in a first step and language (French vs. English) in a second step. They were run for outcome measures where significant cross-language differences were found, that is: the number of words produced in the texts (where English children were more productive than French peers), the proportion of words correct in both tasks (where English children were more accurate than French peers); the rate of morphological errors in texts and dictated words and the rate of semantic errors in the texts only (where French children produced more errors than their English peers); the rate of orthographic and semantic errors in the dictated words (where English children produced more errors than their French peers).

Number of words produced in the texts was the only continuous outcome with normally distributed residuals and a generalized linear model was applied using the lm() function in R (R Core Team, 2018). For all other measures, beta regressions for beta-distributed outcomes were applied, using the betareg() function in R (Cribari-Neto and Zeileis, 2010). Zero-order correlations between all variables of interest are presented in the first instance.

#### Zero-Order Correlations Between the Measures of Interest

Table 6 presents the correlation between the control and spelling measures, for the French and the English samples separately.

**TABLE 6:** Correlation table for the control and spelling variables of interest.

	Age	NVP	PA	Text prod.	12 words acc.	Text acc.	Text MOR	12 words MOR	12 words ORTH	Text SEM	12 words SEM
	French										
Age		0.43	0.12	0.23	0.46*	0.37	–0.15	−0.61***	–0.33	–0.22	–0.12
NVP	0.63***		0.44*	0.39	0.34	0.42	–0.35	−0.48*	–0.20	–0.26	–0.19
PA	0.13	0.59***		0.46*	0.67***	0.54**	–0.09	–0.43	–0.41	−0.53**	–0.01
Text prod.	0.40	0.59***	0.46*		0.58***	0.68***	–0.11	−0.44*	–0.23	−0.53**	–0.21
12 words acc.	0.42	0.70***	0.54**	0.63***		0.73***	–0.20	−0.76***	−0.67***	−0.64***	–0.27
Text acc.	0.54**	0.65***	0.59***	0.59***	0.78***		–0.40	−0.63***	−0.47*	−0.79***	–0.08
Text MOR	–0.22	–0.18	–0.37	–0.08	–0.32	−0.47*		0.39	0.22	0.15	0.17
12 words MOR	–0.34	−0.63***	−0.52**	−0.66***	−0.79***	−0.66***	0.21		0.54**	0.59***	0.22
12 words ORTH	−0.58***	−0.72***	−0.48*	−0.63***	−0.85***	−0.75***	0.39	0.72***		0.43	0.23
Text SEM	0.09	–0.24	–0.26	0.04	–0.29	–0.27	0.15	0.33	0.19		0.03
12 words SEM	–0.10	–0.29	−0.52**	–0.31	−0.51**	–0.36	–0.01	0.36	0.29	0.05	
	English										

Significance levels of Spearman correlations are indicated as follows: *p < 0.05, **p < 0.01, ***p < 0.001, p-values were corrected for multiple tests using Holm–Bonferroni procedure [rcorr.adjust() function in the RcmdrMisc package (Fox, 2018)]; NVP, Non-Verbal Performance, as measures by the Raven’s matrices; PA, Phonological Awareness; Text prod., raw number of words attempted in the texts; 12 words acc., Proportion of words correctly spelled in the 12 words analyzed from the WIAT spelling test; Text acc., Proportion of words correctly spelled in the texts produced using the writing Curriculum-Based Measure; Text MOR, proportion of morphological errors per word within the CBM texts; 12 words MOR, proportion of morphological errors per word within the WIAT 12 words; 12 words ORTH, Proportion of orthographic errors per word within the WIAT 12 words; Text SEM, proportion of semantic errors per words within the CBM texts; 12 words SEM, proportion of semantic errors per words within the WIAT 12 words.

Non-verbal performance and phonological awareness correlated strongly with most of the spelling outcomes selected. In English, both measures correlated strongly with the accuracy measures on both tasks, and with the rate of morphological and orthographic spelling errors in the 12 dictated words in particular. In French, they were also strong correlates with the spelling accuracy and productivity measures (in addition to age). In French, phonological awareness was a strong correlate of semantic errors in texts but did not correlate with semantic errors in the dictated words nor with morphological errors in the texts.

#### Regression Models for Productivity and Accuracy Measures Where English Children Outperformed French Children

Stepwise regressions were run to assess the effect of language over and above age, NVP and phonological awareness, for the productivity and accuracy measures where English children performed better than French children: (1) number of words produced in the texts, (2) proportion of correct words in the texts, (3) proportion of correct words in the 12 dictated words. These regressions are presented in Table 7.

**TABLE 7:** Regression models for productivity and accuracy outcomes where English children outperformed French children.

Outcome: number of words produced in the texts												
	**Model step 1**						**Model step 2**					
	***R*^2^**	***B***	***SE***	***t/F_*R*_^2^***		***p***	***R*^2^**	***B***	***SE***	***t/F_*R*_^2^***		***p***
		
Constant		–30.2	12.4	–2.4		0.017*		–49.8	12.3	–4.05		0.001***
Age		2.10	1.48	1.42		0.159		3.12	1.38	2.25		0.026*
NVP		1.02	0.53	2.04		0.044*		0.91	0.049	1.84		0.069
PA		0.52	0.37	1.41		0.161		0.94	0.35	2.69		0.008**
Lang (EN)								14.98	3.48	4.31		< 0.001***
Model	0.182			8.433		< 0.001***	0.308			12.1		< 0.001***
*R*^2^ change							0.125			18.54		< 0.001***

**Outcome: proportion of words correct in the texts**												

	**Model step 1**						**Model step 2**					
	***R*^2^**	***B***	***SE***	***χ^2^***	***df***	***p***	***R*^2^**	***B***	***SE***	***χ^2^***	***df***	***p***
		
Constant		–2.33	0.74	–3.13	1	0.0017**		–4.75	0.72	–6.6	1	< 0.001***
Age		0.09	0.09	1.02	1	0.31		0.22	0.08	2.83	1	0.005**
NVP		0.06	0.03	2.03	1	0.043*		0.05	0.03	1.68	1	0.09
PA		0.01	0.02	0.63	1	0.527		0.07	0.02	3.44	1	0.0005***
Lang (EN)								1.59	0.21	7.67	1	< 0.001***
Model	0.147			21.53	5		0.478			45.74	6	
*R*^2^ change							0.330			58.85		< 0.001***

**Outcome: proportion of words correct in the 12 dictated words**												

	**Model step 1**						**Model step 2**					
Constant		–4.93	0.75	–6.60	1	< 0.001***		–6.97	0.75	–9.25	1	< 0.001***
Age		0.24	0.08	2.79	1	0.005**		0.34	0.08	4.304	1	< 0.001***
NVP		0.04	0.03	1.41	1	0.16		0.03	0.03	1.11	1	0.266
PA		0.07	0.02	3.14	1	0.0017**		0.11	0.02	5.41	1	< 0.001***
Lang (EN)								1.28	0.20	6.39	1	< 0.001***
Model	0.245			20.57	5		0.445			39.5	6	
*R*^2^ change							0.200			40.84		< 0.001***

##### Number of words produced in the texts

The initial model with age, NVP and phonological awareness explained a significant 18.52% of variance in text productivity. The addition of the language predictor in a second step explained a significant further 12.52% of variance (new model *R*^2^ = 30.75%).

##### Proportion of words correct in the texts

The initial model with age, NVP and phonological awareness explained 14.73% of variation in the proportion of correct words in the texts. The addition of language in a second step explained a significant further 33.02% (new model *R*^2^ = 47.74%).

##### Proportion of words correct in the 12 dictated words

The initial model with age, NVP and phonological awareness explained 24.49% of variation in the proportion of correct words in the 12 dictated words. The addition of language in a second step explained a significant further 20.02% (new model *R*^2^ = 44.51%).

The regressions confirmed language was a significant predictor of our productivity and accuracy measures of interest, over and above age, NVP and phonological awareness. All control measures being equivalent, English students were more likely than French students to produce longer and more accurate texts, and more correct words in the dictated words.

#### Regression Model for the Qualitative Outcome Measures Where English Children Outperformed French Children

Stepwise regressions were run to assess the effect of language over and above age, NVP and phonological awareness, for the qualitative outcome measures where English children performed better than French children: (1) morphological errors in the texts, (2) morphological errors in the 12 dictated words, (3) semantic errors in the texts. These regressions are presented in Table 8.

**TABLE 8:** Regression models for qualitative outcome measures where English children outperformed French children.

**Outcome: proportion of morphological errors in the texts**												
	**Model step 1**						**Model step 2**					
	***R*^2^**	***B***	***SE***	***χ^2^***	***df***	***p***	***R*^2^**	***B***	***SE***	***χ^2^***	***df***	***p***
		
Constant		–2.1	0.67	–3.17	1	0.001**		–0.50	0.63	–0.79	1	0.43
Age		0.03	0.08	0.36	1	0.72		–0.06	0.07	–0.88	1	0.38
NVP		–0.02	0.03	–0.78	1	0.43		–0.02	0.02	–0.80	1	0.42
PA		0.03	0.02	1.26	1	0.21		0.01	0.02	0.43	1	0.66
Lang (EN)								–1.44	0.19	–7.55	1	< 0.001***
Model	0.016			128.4	5		0.478			153.5	6	
*R*^2^ change							0.320			56.9		< 0.001***

**Outcome: proportion of morphological errors in the 12 dictated**												

	**Model step 1**						**Model step 2**					
Constant		2.07	0.59	3.49	1	< 0.001***		3.75	0.57	6.56	1	< 0.001***
Age		–0.09	0.07	–1.22	1	0.22		–0.21	0.06	–3.42	1	< 0.001***
NVP		–0.08	0.03	–3.23	1	0.001**		–0.06	0.02	–2.75	1	0.006**
PA		–0.01	0.02	–0.81	1	0.42		–0.05	0.02	–3.10	1	0.002**
Lang (EN)								–1.21	0.17	–7.19	1	< 0.001***
Model	0.222			74.01	5		0.476			94.93	6	
*R*^2^ change							0.254			51.69		< 0.001***

**Outcome: proportion of semantic errors in the texts**												

	**Model step 1**						**Model step 2**					
Constant		–1.43	0.70	–2.04	1	0.04*		0.24	0.72	0.34	1	0.74
Age		0.11	0.08	1.32	1	0.18		0.02	0.08	0.29	1	0.77
NVP		–0.06	0.03	–1.98	1	0.048*		–0.05	0.03	–1.85	1	0.06
PA		0.01	0.02	0.23	1	0.82		–0.03	0.02	–1.41	1	0.16
Lang (EN)								–1.05	0.21	–5.01	1	< 0.001***
Model	0.0627			148.5	5		0.369			159	6	
*R*^2^ change										25.11	1	< 0.001***

##### Proportion of morphological errors in the texts

The initial model with age, NVP and phonological awareness explained 1.58% of variation in the rate of morphological errors in the texts. The addition of language in a second step explained a significant further 46.18% (new model *R*^2^ = 47.76%).

##### Proportion of morphological errors in the 12 dictated words

The initial model with age, NVP and phonological awareness explained 22.23% of variation in the rate of morphological errors in the 12 dictated words. The addition of language in a second step explained a significant further 25.37% (new model *R*^2^ = 47.6%).

##### Proportion of semantic errors in the texts

The initial model with age, NVP and phonological awareness explained 6.27% of variation in the rate of semantic errors in the 12 dictated words. The addition of language in a second step explained a further 30.67% (new model *R*^2^ = 36.94%).

The regressions confirmed language was a significant predictor of our qualitative measures of interest, over and above age, NVP and phonological awareness. English children were less likely than French children to produce morphological and semantic errors in the texts, and morphological errors in the dictated words, regardless of age, NVP and phonological awareness levels.

#### Regression Model for the Qualitative Outcome Measures Where French Children Outperformed English Children

Stepwise regressions were run to assess the effect of language over and above age, NVP and phonological awareness, for the qualitative outcome measures where French children performed better than English children: (1) orthographic errors in the 12 dictated words, (2) semantic errors in the 12 dictated words. The regression models for these outcome measures are presented in Table 9.

**TABLE 9:** Regression models for qualitative outcome measures where French children outperformed English children.

**Outcome: proportion of orthographic errors in the 12 dictated words**												
	**Model step 1**						**Model step 2**					
	***R*^2^**	***B***	***SE***	***χ^2^***	***df***	***p***	***R*^2^**	***B***	***SE***	***χ^2^***	***df***	***p***
		
Constant		5.51	0.72	7.62	1	< 0.001***		5.43	0.77	7.06	1	< 0.001***
Age		–0.31	0.08	–3.78	1	< 0.001***		–0.31	0.08	–3.67	1	< 0.001***
NVP		–0.07	0.03	–2.40	1	0.017*		–0.07	0.03	–2.45	1	0.014*
PA		–0.05	0.02	–2.65	1	0.008**		–0.05	0.02	–2.43	1	0.015*
Lang (EN)								0.07	0.20	0.35	1	0.72
Model	0.362			30.19	5		0.361			30.25	6	
*R*^2^ change							–0.07			0.13		0.72

**Outcome: proportion of semantic errors in the 12 dictated words**												

	**Model step 1**						**Model step 2**					
Constant		–1.11	0.58	–1.92	1	0.055		–2.06	0.60	–3.44	1	< 0.001***
Age		–0.09	0.07	–1.34	1	0.18		–0.05	0.07	–0.82	1	0.41
NVP		0.01	0.03	0.24	1	0.81		0.005	0.02	0.21	1	0.84
PA		–0.07	0.02	–4.14	1	< 0.001***		–0.06	0.02	–3.66	1	< 0.001***
Lang (EN)								0.76	0.18	4.18	1	< 0.001***
Model	0.212			225.4	5		0.370			234.2	6	
*R*^2^ change							0.158			17.51	1	< 0.001***

##### Proportion of orthographic errors in the 12 dictated words

The initial model with age, NVP and phonological awareness explained 36.15% of variation in the rate of orthographic errors in the 12 dictated words. The addition of language in a second step reduced the model’s prediction coefficient (Pseudo *R*^2^ = 36.08%).

##### Proportion of semantic errors in the 12 dictated words

The initial model with age, NVP and phonological awareness explained 21.2% of variation in the rate of semantic errors in the 12 dictated words. The addition of language in a second step explained a significant further 25.37% (new model *R*^2^ = 47.6%).

The regressions confirmed the importance of language in explaining the proportion of semantic errors, over and above age, NVP and phonological awareness. With equivalent age, NVP scores and phonological awareness scores, French students were less likely than English students to produce semantic errors in the dictated words. However, language was not a significant contributor to the model explaining the proportion of orthographic errors in the 12 words.

## Discussion

The present study aimed to characterize the spelling difficulties of children with DLD at the end of primary school, in two languages of similar orthographic opacity, but contrasted for their linguistic constraints: French and English. The results point to cross-language differences in text productivity and error rates, with all French groups producing shorter and less accurate texts than their English peers overall. They also point to qualitative differences in the locus of these errors, with more orthographic errors in the English dictation samples and more morphological errors in the French texts and dictation samples. Nevertheless, across languages and error types, children with DLD performed in line with their SA but not CA peers, suggesting a delay in their spelling profiles commensurate with language and literacy levels. Fine-grained analysis of errors further shows language-specific constraints in the spelling of each group of children.

### Word- and Sentence- Level Processes Involved in Spelling Across French and English

By using a linguistic framework for the assessment of spelling errors, we were able to highlight differences in the constraints affecting spelling in French and English. It was predicted that orthographic constraints were more likely to affect spelling performance in English, whilst morphological constraints were more likely to affect spelling performance in French. We indeed found poorer morphological spelling scores in French as compared to English, in both tasks, but we could not quite highlight any difference in the rate of orthographic errors between the two languages, in any of the tasks, although the proportion of orthographic errors in dictation was slightly higher in English than French altogether. This result highlights the importance of considering spelling as a multi-component skill rather than as a single construct, with lexical and sublexical constraints on the one hand, and grammatical constraints on the other (Morin et al., 2018). It also emphasizes the need for several tasks to tap into these distinct mechanisms. The assessment of spelling is often limited to word-level tasks, emphasizing the influence of word properties, such as syllabic complexity, frequency and transparency, on spelling performance (Wimmer and Landerl, 1997; Marinelli et al., 2015). In our study, the orthographic constraints of English appeared only in the word dictation task, where children could not choose the words they spelled, whilst French morphological constraints were most evident in text production, where children had to consider the grammatical context of many words in order to spell inflections accurately. Finally, the many segmentation errors found in the French younger and DLD samples were evidenced in text production only. To our knowledge, the present study provides the first direct comparison of word- as well as sentence-level constraints on spelling in English compared to another language. It was striking that our French sample overall produced shorter and less accurate texts than their English peers, despite English being consistently described as an outlier in terms of spelling difficulty (Share, 2008). We argue that future studies of spelling development are needed, that contrast orthographies not only for orthographic consistency but also morphological richness, both derivational and inflectional (see for example Desrochers et al., 2018, contrasting English, French, and Greek on these aspects).

### Developmental Patterns of Spelling in Children With DLD

The present work was motivated by a meta-analytic review of the literature on the spelling performance of children with DLD across European orthographies (Joye et al., 2019). That review did not highlight any difference in the quantity of errors produced by children with DLD compared to younger typically developing children matched for language or literacy skills, but did highlight a clear lag in spelling scores compared to same-age children. By comparing the spelling errors of these three groups of children qualitatively, we aimed to assess whether the locus of these spelling difficulties might differ in children with DLD when a more detailed analysis of their spelling errors was included.

Our group comparisons did not highlight significant differences in the spelling profiles of children with DLD and younger typically developing children matched for spelling level. Children with DLD produced errors similar to those of their younger peers, and in similar proportions, that is: segmentation errors in French texts, errors with contextual patterns and inconsistent vowel spellings in English, and a range of phonological errors in both languages. Errors with inflection omissions and contractions were also found in the English SA and DLD samples, whilst in French, morpheme substitutions were most common, and found overwhelmingly across all three groups.

It should be noted that all comparisons were run with a stringent significance threshold of 0.005, due to the multiple comparisons being conducted in the study (i.e., Holm–Bonferroni correction for multiple comparisons). Visual examination of the data (Figure 1) does suggest that French children with DLD might produce a slightly higher rate of phonological errors than their SA peers. It also suggests the distribution for this error type is spread toward the higher end in the English-DLD sample as compared to their SA peers. On the other hand, orthographic and morphological errors seem to be slightly higher in the younger group in both languages (than both CA and DLD groups). Considered together, these visual trends suggest a developmental pattern whereby children with DLD are delayed in their orthographic and morphological spelling, but might remain more impaired than should be expected in the phonological domain. However, these trends are not corroborated by the numerical comparisons.

The current results also provide developmental benchmarks for the assessment of spelling in a population of children with DLD in French and English middle school. Future studies may want to characterize further the spelling profiles of French and English children in early primary and secondary school. This has been done to an extent for adolescents in previous studies (Dockrell et al., 2009; Broc et al., 2013, 2014), but those studies have not included a younger group of SA TD matched peers, making it difficult to assess whether patterns of spelling development are typical in the population of children with DLD over time. Further data are needed to test whether morphological ending errors appear in French samples later on in adolescence, and to what extent they deviate from younger peers matched for spelling level. Future studies may also explore whether morphological ending and contraction errors (in English) and segmentation errors (in French), as well as phonological errors (in both languages), persist in adolescence, over and above what might be expected given overall spelling development. Longitudinal designs may also be appropriate for this type of characterization (Nauclér, 2004).

### Linguistic Constraints in the Spelling Development of Children With DLD

The phonological and morphological difficulties of children with DLD have often been investigated in their early oral language (Leonard, 2014). One aim of the current study was to assess whether some of these oral language difficulties remain in written language, and to assess whether they could be found in languages other than English and thus test any claim for universality in atypical language development. A few studies have found suffix omissions to be a particular feature of the spelling of children with DLD (Windsor et al., 2000; Mackie and Dockrell, 2004; Silliman et al., 2006; Larkin et al., 2013; Mackie et al., 2013; Critten et al., 2014). Those studies were conducted in English only, and pointed to specific difficulties with spelling -*ing*, plural and 3rd person *-s* and past tense *-ed*, as also observed in the oral language of English children with DLD (Windsor et al., 2000). In our analysis, these errors were classified as “omissions of a morphological marker affecting phonology,” within the morphological category. Observation of the descriptive data as shown in Appendices E, F suggests that these errors are also found overwhelmingly in our English DLD sample. However, they are not found in the French DLD and SA samples. Instead, a large number of segmentation errors were found in the French DLD and SA samples, either in the semantic category (“Segmentation errors,” e.g., ^∗^les cole for l’école) or in the morphological contraction category (“Errors on word contractions,” e.g., ^∗^quon for qu’on).

Several interpretations can be drawn from these data. Firstly, our data suggest that, if specific to the population of *English* children with DLD, inflection omission errors are not necessarily found in other languages, at least not in French middle school, arguing against any claim for universality of these particular error types. Secondly, our data question whether the drivers of these specific errors in English are morphological in nature. It has been argued that phonological salience is an important factor to consider when assessing morphological omission errors in the oral language of children with DLD (Parisse and Maillart, 2007). It is possible that English children with DLD continue to produce omissions of non-salient morphological markers for an extended period of time, just as they continue producing other errors with difficult phonological combinations (such as consonant cluster reductions, substitutions of closely related consonants and vowels) in both French and English. Finally, our written data complements accounts of French DLD-specific errors in oral language (Jakubowicz et al., 1998; Hamann et al., 2003; Thordardottir and Namazi, 2007). In oral language, French clitic pronouns have been found to be particularly difficult for children with DLD. We found here that those speech segments which can be found in multiple lexical and grammatical contexts, and present with some degree of phonetic similarity (la, le, les, me, m’, te, t’, etc.) continue to be difficult to represent in written language for French children with DLD in late primary school, with a high rate of morphological errors in the contraction category (see Appendices E, F for a breakdown of error types). These errors, along the many segmentation errors found in the French DLD and SA sample (e.g., récré a tion), highlight the immature lexical representations of this population, and suggest difficulties integrating grammatical information from non-phonologically salient units. This is in line with evidence from typical development suggesting phonological and non-phonological aspects of language are intricately related in the early years (Hipfner-Boucher et al., 2014), and evidence from children with DLD highlighting their sensitivity to phonological aspects of grammatical segments (Tomas et al., 2015). It is likely that difficulties with both segmental and supra-segmental aspects of language in this population drive further difficulties with later lexical and orthographic representations (Share and Shalev, 2004). Our data do not settle this matter, but do suggest that linguistic constraints apply to written as well as oral language, and that assessing spelling qualitatively does indeed provide a good “window into residual language deficits” (Bishop and Clarkson, 2003), and possibly the representation of phonetically subtle but grammatically discrete linguistic units Of course, spelling is not just about phonology and morphology, and involves a range of orthographic constraints that children need to learn and apply in their production. Our linguistic framework for spelling error analysis also incorporates these constraints and shows how much they indeed influence the spelling performance of children with DLD, in support of previous studies (Soriano-Ferrer and Contreras-González, 2012). We argue that a broad linguistic framework incorporating oral as well and written word forms is likely to be appropriate to assess and support the spelling development of children with DLD (Apel and Masterson, 2001).

### Underlying Processes in French and English Spelling Development: Commonalities and Differences

Possible constraints in understanding the spelling profiles of our sample were explored with a set of control measures (age, non-verbal performance and phonological awareness).

Overall, the regression analyses supported the role of language found in the quantitative and qualitative errors analyses conducted in sections “Productivity and accuracy within and across languages” and “Qualitative analysis of spelling errors.” Even after accounting for age, NVP and phonological awareness, language was still a significant predictor of text productivity and spelling accuracy in the text and dictated words. English children were more likely to obtain higher scores on these measures, but also more likely than French children to produce semantic errors in the dictated words. By contrast, French children were more likely to produce a higher proportion of morphological and semantic errors in their texts and morphological errors in the dictated words. There was one exception to these confirmatory results: language did not predict the proportion of orthographic errors in the 12 dictated words, over and above age, NVP and phonological awareness. In this model, age was a particularly good predictor of the decrease in the proportion of orthographic errors, suggesting in both languages, continued exposure with written content improves the retention of orthographic patterns, in line with self-teaching accounts (Conrad, 2008; Shahar-Yames and Share, 2008).

The correlations presented in section “Zero-order correlations between the measures of interest” also provided some insight into the processes involved in different components of spelling in the two languages. Age, NVP and phonological awareness correlated with most of the spelling outcomes considered, in both languages. However, in French, the proportion of semantic errors in the dictated words and morphological errors in the texts was not associated with age or phonological awareness. We interpret this as an indication that these errors are related to spelling skills that were still not mastered in the French older control group (homophones and morphological inflections). In contrast, large correlations were found between phonological awareness and semantic errors in the texts, and morphological/orthographic errors in the 12 dictated words, indicating phonological awareness plays an important role in representing semantic, orthographic and morphological units in French word spelling. In English, all three control measures correlated with the spelling productivity and accuracy variables and most qualitative measures. However, semantic errors in general and morphological errors in the texts did not correlate very strongly with the predictors. This likely reflects the low rate of such errors in the English sample altogether.

Phonological skills have been related to French and English spelling in previous studies (Moll et al., 2014), and to an extent, our models confirmed previous findings: (1) English spelling is particularly reliant on phono-graphemic skills, and possibly for an extended period of time than other orthographies, due to its opacity; (2) French spelling is also reliant on phonological skills early on, but may also call on a wider range of processes later on (see Moll et al., 2014; Desrochers et al., 2018). However, in the existing literature, spelling had been considered as a single construct. By differentiating between different component spelling skills in the present study, we also found differentiated patterns of relationships. This was rather an incidental finding as the focus of the present study was really on spelling errors. Future studies may want to further investigate the nature of the differences observed in the present study, including predictors of different components of text spelling such as morphological awareness (e.g., in Desrochers et al., 2018) but also reading and transcription skills involved at different stages of the development of text production (Llaurado and Dockrell, 2020). We also suggest that reading might be a better indicator of phonological skills in late primary school than an explicit measure of phonological awareness. Not all children with DLD in our sample presented with low phonological awareness scores, but a large proportion of them presented with low reading scores (as shown in Table 1).

### Limitations

Although the present study attempted to draw on a range of linguistic components in the list of words that children were given to spell, it was impossible to match our French and English list of words on all sublexical aspects critical to spelling (morphological, orthographic and phonological complexity as well as frequency, number of orthographic and phonological neighbors, syllabic complexity and word length). Arguably, 12 words is also too small a list to be fully representative of the constraints of each language. Spelling error analysis is a time-consuming process requiring several raters and several rounds of coding, for adequate training and reliability checks. Unfortunately, because of constraints with time and raters’ availability, we had to restrict the analysis to a limited number of words, which we attempted to match across languages. Such attempts are of course imperfect. Future studies may, however, rely on recent developments in the cross-language assessment of language and literacy. One promising tool for future analyses is the Multilanguage Assessment Battery of Early Literacy, recently made available online for a range of languages (MABEL, Caravolas et al., 2020) and which authors may want to consider when developing cross-language studies of spelling errors.

Similarly, giving children a free writing task does not allow to capture all the spelling processes that may be at play in spelling for writing. Children may or may not use some of the spelling processes we were aiming to assess. Arguably as well, 5 min is a rather short amount of time for children to generate ideas and produce a text, and it is likely that children’s familiarity with this type of tasks may have affected their productivity and spelling performance on this task. Observations during data collection and discussions with teachers in both French contexts suggest that French children are typically given more time and scaffolding in writing tasks and may have been surprised by such a short and free writing task. Such accounts were not given in the English contexts.

Beyond task differences, cross-language comparisons between French and English are complicated by the fact children do not start formal literacy instruction at the same age. Although as a group, the age of the French and English samples did not differ significantly, English children had typically been in formal education for a year longer than their French peers. One cannot rule out the possibility that this difference affected our cross-language comparisons. Socio-economic and instructional factors could also be controlled in future. It is possible that the explicit teaching (or the lack of) of particular aspects of spelling could affect children’s response and the quality of their errors in our spelling tasks.

## Conclusion

The present study assessed the locus of spelling difficulties in two samples of middle school children with DLD in France and in the United Kingdom. Results suggest a pattern of overall delay in spelling development in children with DLD in both languages, with a range of phonological, orthographic, morphological and semantic errors similar to those of younger peers. They also confirm that the difficulties observed in the early oral language of children with DLD persevere in late primary school in written language, that is difficulties with morphological endings in English and difficulties with pronoun contractions and word segmentation in French. We argue that these specific difficulties in each language might be related to the phonological salience of these grammatical forms. In the general population too, error types were specific to each language assessed, with clear orthographic constraints in English, and morphological constraints in French. Further studies may want to assess children’s spelling at other developmental timepoints and in a broader range of languages contrasted for phono-orthographic transparency and morphological complexity.

## Data Availability Statement

The datasets generated for this study are available on request to the corresponding author.

## Ethics Statement

This study involved human participants and was approved by the ethics committee of UCL Institute of Education. Written informed consent to participate in this study was provided by the participants and by their legal guardian/next of kin.

## Author Contributions

NJ planned, collected the data, and analyzed and wrote for the present study. JD and CM advised and provided feedback at all of these research stages. All the authors contributed to the article and approved the submitted version.

## Conflict of Interest

The authors declare that the research was conducted in the absence of any commercial or financial relationships that could be construed as a potential conflict of interest.
