# Supplementary material for: The Spelling Errors of French and English Children With Developmental Language Disorder at the End of Primary School
Source: Front Psychol. 2020 Jul 21;11:1789. doi: 10.3389/fpsyg.2020.01789 (PMC7386207; doi:10.3389/fpsyg.2020.01789)
Supplement: Supplementary file 1 [file Data_Sheet_1.docx]

## Appendix A: Fine-grained count of phonological errors in texts


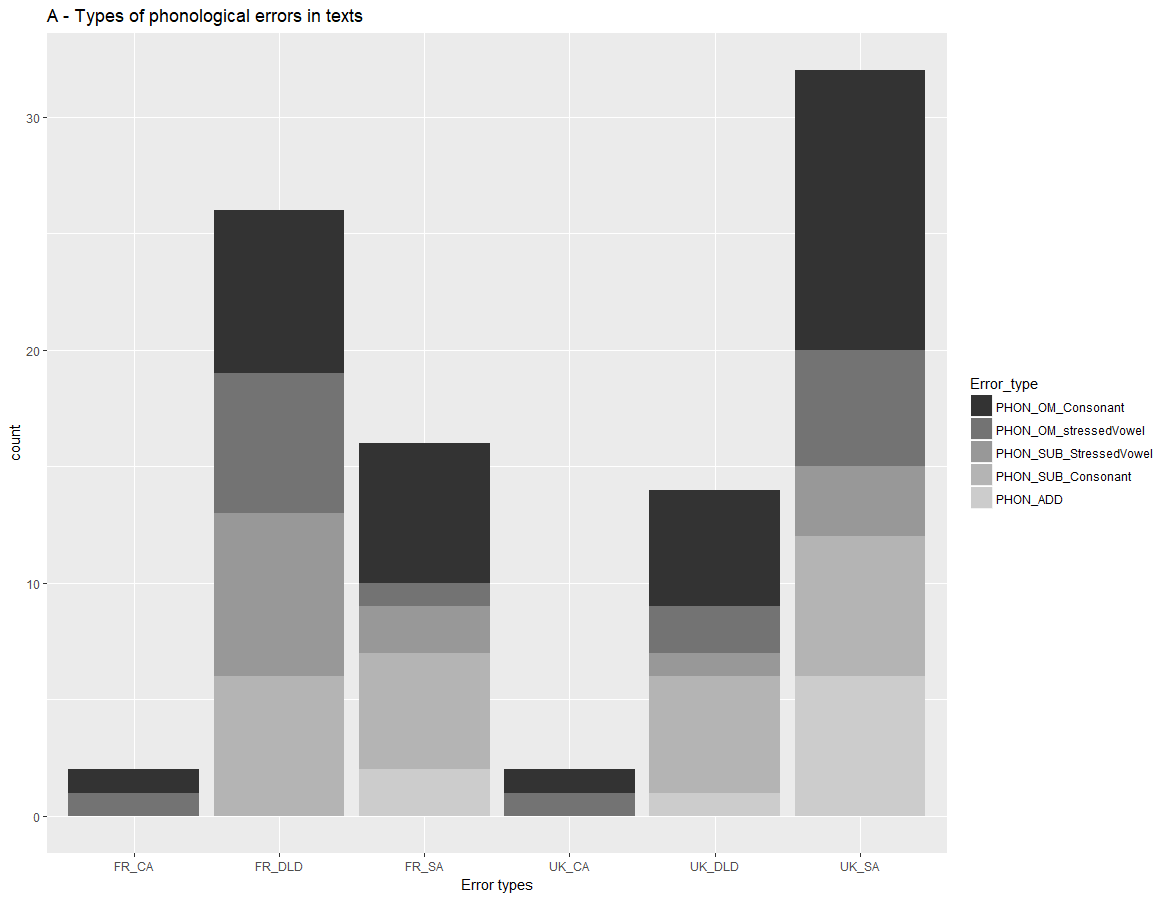


|  | FR_CA | FR_DLD | FR_SA | UK_CA | UK_DLD | UK_SA |
| --- | --- | --- | --- | --- | --- | --- |
| PHON_OM_Consonant | 1 | 7 | 6 | 1 | 5 | 12 |
| PHON_OM_stressedVowel | 1 | 6 | 1 | 1 | 2 | 5 |
| PHON_SUB_StressedVowel | 0 | 7 | 2 | 0 | 1 | 3 |
| PHON_SUB_Consonant | 0 | 6 | 5 | 0 | 5 | 6 |
| PHON_ADD | 0 | 0 | 2 | 0 | 1 | 6 |

*Notes.* PHON-OM-Consonant: Omission of an obligatory consonant, PHON-OM-stressedVowel: Omission of a stressed vowel, PHON-SUB-StressedVowel: Substitution of a stressed vowel, PHON-SUB-Consonant: Substitution of a consonant, PHON-ADD: Addition of a phoneme

# Appendix B: Fine-grained count of phonological errors in 12 dictated words


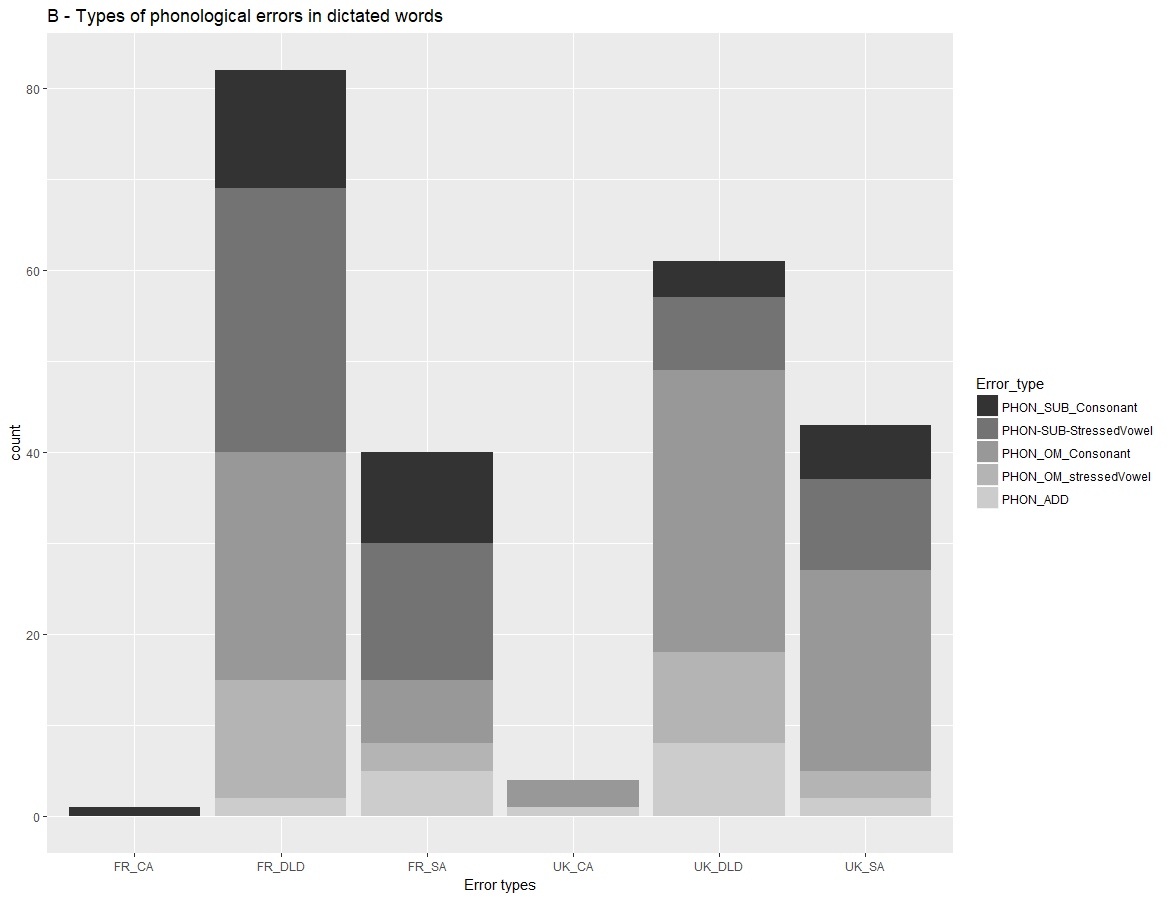


|  | FR_CA | FR_DLD | FR_SA | UK_CA | UK_DLD | UK_SA |
| --- | --- | --- | --- | --- | --- | --- |
| PHON_SUB_Consonant | 1 | 13 | 10 | 0 | 4 | 6 |
| PHON-SUB-StressedVowel | 0 | 29 | 15 | 0 | 8 | 10 |
| PHON_OM_Consonant | 0 | 25 | 7 | 3 | 31 | 22 |
| PHON_OM_stressedVowel | 0 | 13 | 3 | 0 | 10 | 3 |
| PHON_ADD | 0 | 2 | 5 | 1 | 8 | 2 |

*Notes*. PHON-SUB-Consonant: Substitution of a consonant, PHON-SUB-StressedVowel: Substitution of a stressed vowel, PHON-OM-Consonant: Omission of an obligatory consonant, PHON-OM-stressedVowel: Omission of a stressed vowel, PHON-ADD: Addition of a phoneme

# Appendix C: Fine-grained count of orthographic errors in texts


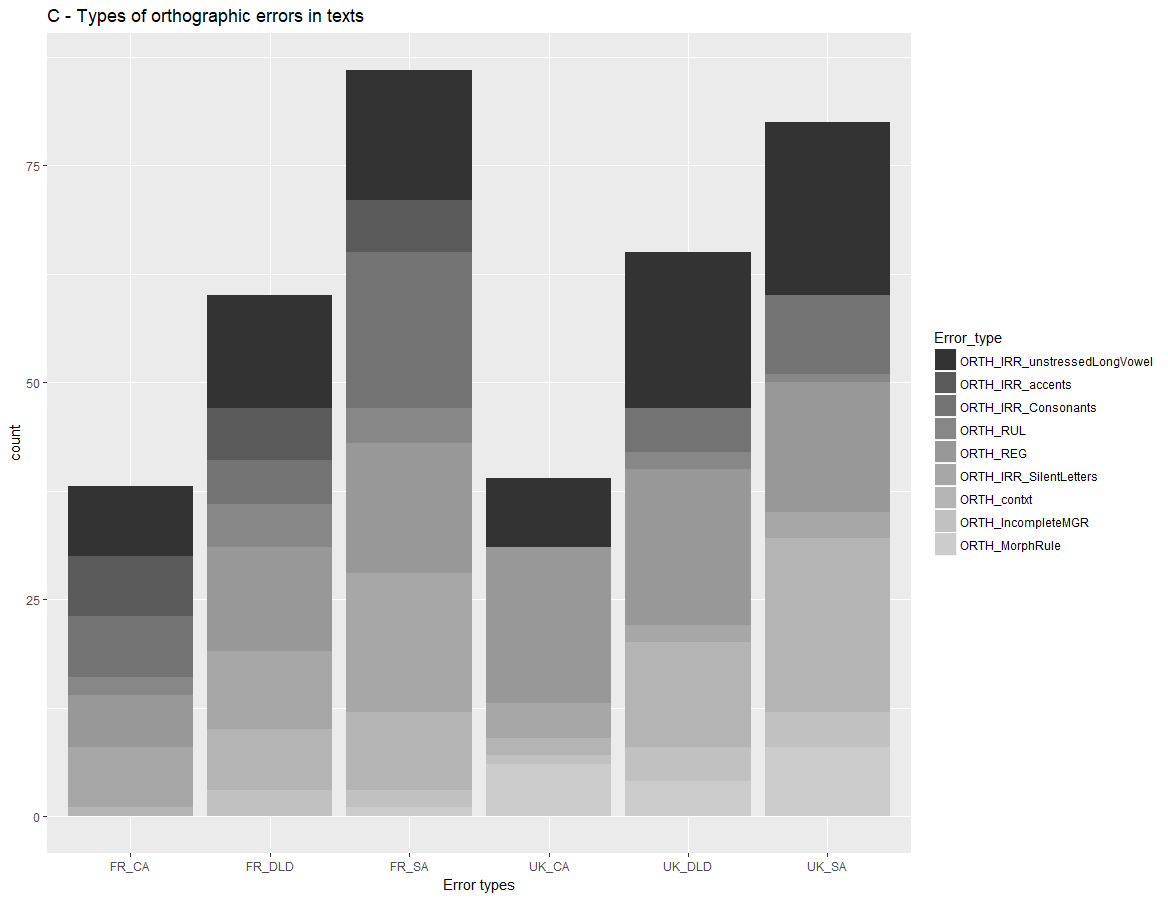


|  | FR_CA | FR_DLD | FR_SA | UK_CA | UK_DLD | UK_SA |
| --- | --- | --- | --- | --- | --- | --- |
| ORTH_IRR_unstressedLongVowel | 8 | 13 | 15 | 8 | 18 | 20 |
| ORTH_IRR_accents | 7 | 6 | 6 | 0 | 0 | 0 |
| ORTH_IRR_Consonants | 7 | 5 | 18 | 0 | 5 | 9 |
| ORTH_RUL | 2 | 5 | 4 | 0 | 2 | 1 |
| ORTH_REG | 6 | 12 | 15 | 18 | 18 | 15 |
| ORTH_IRR_SilentLetters | 7 | 9 | 16 | 4 | 2 | 3 |
| ORTH_contxt | 1 | 7 | 9 | 2 | 12 | 20 |
| ORTH_IncompleteMGR | 0 | 3 | 2 | 1 | 4 | 4 |
| ORTH_MorphRule | 0 | 0 | 1 | 6 | 4 | 8 |

*Notes*. ORTH-IRR- unstressedLongVowel: Substitution or omission of an unstressed vowel grapheme, ORTH-IRR-accent: Error on an accent, ORTH-IRR-Consonants: Substitution of an ambiguous consonant spelling, ORTH-RUL: Error on a taught spelling rule or an illegal letter sequence, ORTH-REG: Error on a regular spelling pattern, ORTH-IRR-SilentLetters: Omission of an unpredictable silent letter, ORTH-contxt: Error with orthographically-constrained graphemes-phoneme correspondences affecting phonology, ORTH-IncompleteMGR: Error of letter inversion, ORTH-MorphRule: Error with rule-constrained applications of inflections and derivations

# Appendix D: Fine-grained count of orthographic errors in 12 dictated words


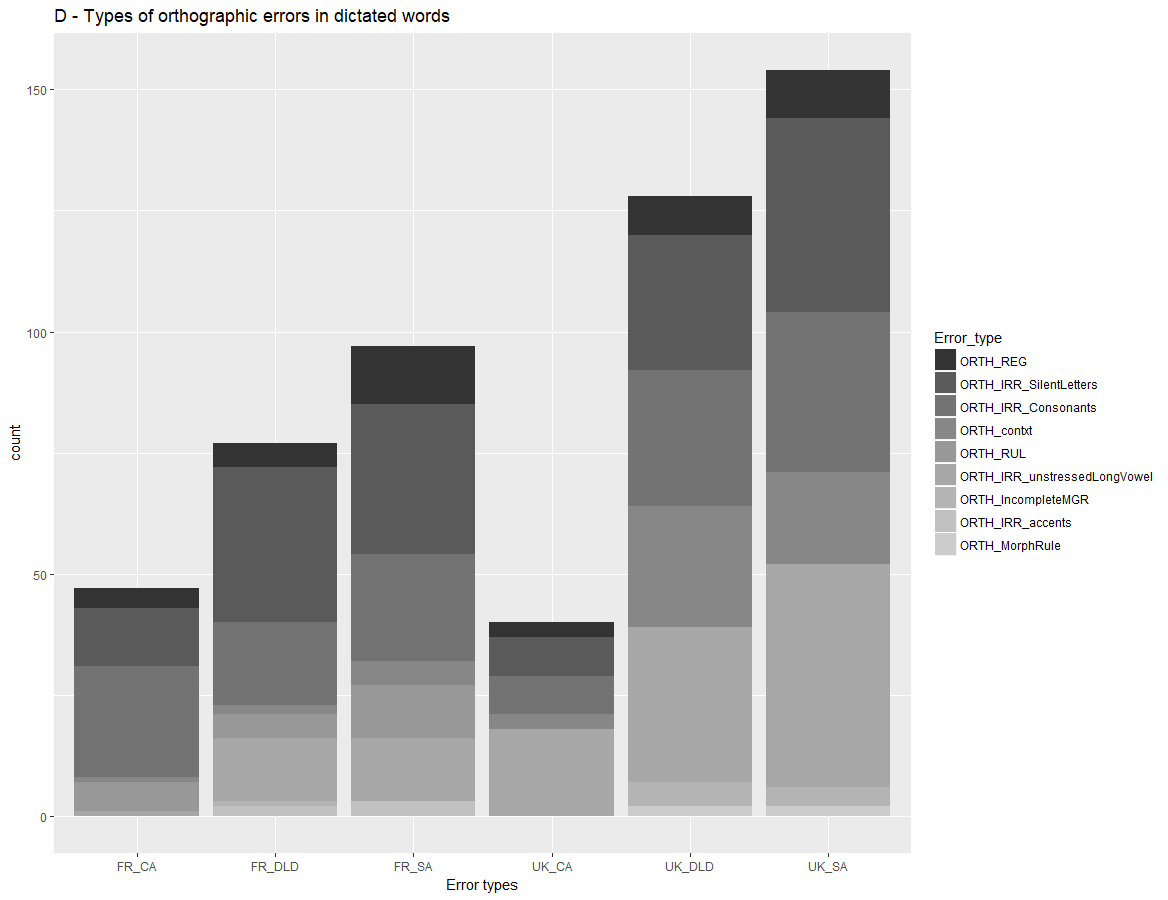


|  | FR_CA | FR_DLD | FR_SA | UK_CA | UK_DLD | UK_SA |
| --- | --- | --- | --- | --- | --- | --- |
| ORTH_REG | 4 | 5 | 12 | 3 | 8 | 10 |
| ORTH_IRR_SilentLetters | 12 | 32 | 31 | 8 | 28 | 40 |
| ORTH_IRR_Consonants | 23 | 17 | 22 | 8 | 28 | 33 |
| ORTH_contxt | 1 | 2 | 5 | 3 | 25 | 19 |
| ORTH_RUL | 6 | 5 | 11 | 0 | 0 | 0 |
| ORTH_IRR_unstressedLongVowel | 1 | 13 | 13 | 18 | 32 | 46 |
| ORTH_IncompleteMGR | 0 | 1 | 0 | 0 | 5 | 4 |
| ORTH_IRR_accents | 0 | 2 | 3 | 0 | 0 | 0 |
| ORTH_MorphRule | 0 | 0 | 0 | 0 | 2 | 2 |

*Notes*. ORTH-REG: Error on a regular spelling pattern, ORTH-IRR-SilentLetters: Omission of an unpredictable silent letter, ORTH-IRR-Consonants: Substitution of an ambiguous consonant spelling, ORTH-contxt: Error with orthographically-constrained graphemes-phoneme correspondences affecting phonology, ORTH-RUL: Error on a taught spelling rule or an illegal letter sequence, ORTH-IRR- unstressedLongVowel: Substitution or omission of an unstressed vowel grapheme, ORTH-IncompleteMGR: Error of letter inversion, ORTH-IRR-accent: Error on an accent, ORTH-MorphRule: Error with rule-constrained applications of inflections and derivations

# Appendix E: Fine-grained count of morphological errors in texts


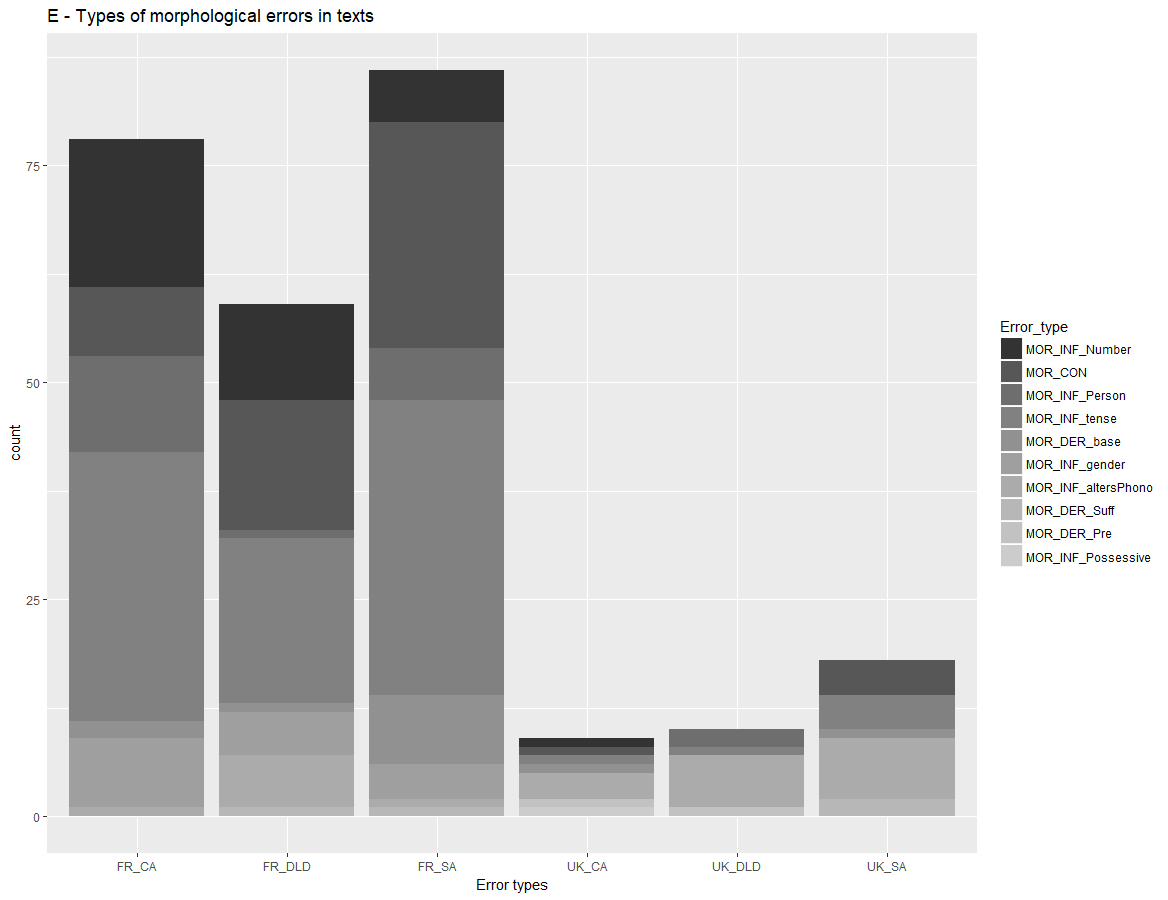


|  | FR_CA | FR_DLD | FR_SA | UK_CA | UK_DLD | UK_SA |
| --- | --- | --- | --- | --- | --- | --- |
| MOR_INF_Number | 17 | 11 | 6 | 1 | 0 | 0 |
| MOR_CON | 8 | 15 | 26 | 1 | 0 | 4 |
| MOR_INF_Person | 11 | 1 | 6 | 0 | 2 | 0 |
| MOR_INF_tense | 31 | 19 | 34 | 1 | 1 | 4 |
| MOR_DER_base | 2 | 1 | 8 | 1 | 0 | 1 |
| MOR_INF_gender | 8 | 5 | 4 | 0 | 0 | 0 |
| MOR_INF_altersPhono | 1 | 6 | 1 | 3 | 6 | 7 |
| MOR_DER_Suff | 0 | 1 | 1 | 0 | 0 | 2 |
| MOR_DER_Pre | 0 | 0 | 0 | 1 | 1 | 0 |
| MOR_INF_Possessive | 0 | 0 | 0 | 1 | 0 | 0 |

*Notes*. MOR-INF-Number: Error on number marking, MOR-CON: Errors on word contractions, MOR-INF- Person: Error on person marking, MOR-INF-tense: Error on tense inflection, MOR-DER-base: Error on the base of a complex word, MOR-INF-gender: Error on gender inflection, MOR-INF-altersPhono: Omission of a morphological marker affecting phonology, MOR-DER-Suff: Error on the suffix of a complex word, MOR-DER-Pre: Error on the prefix of a complex word, MOR-INF-Poss: Error on possessive marking

# Appendix F: Fine-grained count of morphological errors in 12 dictated words


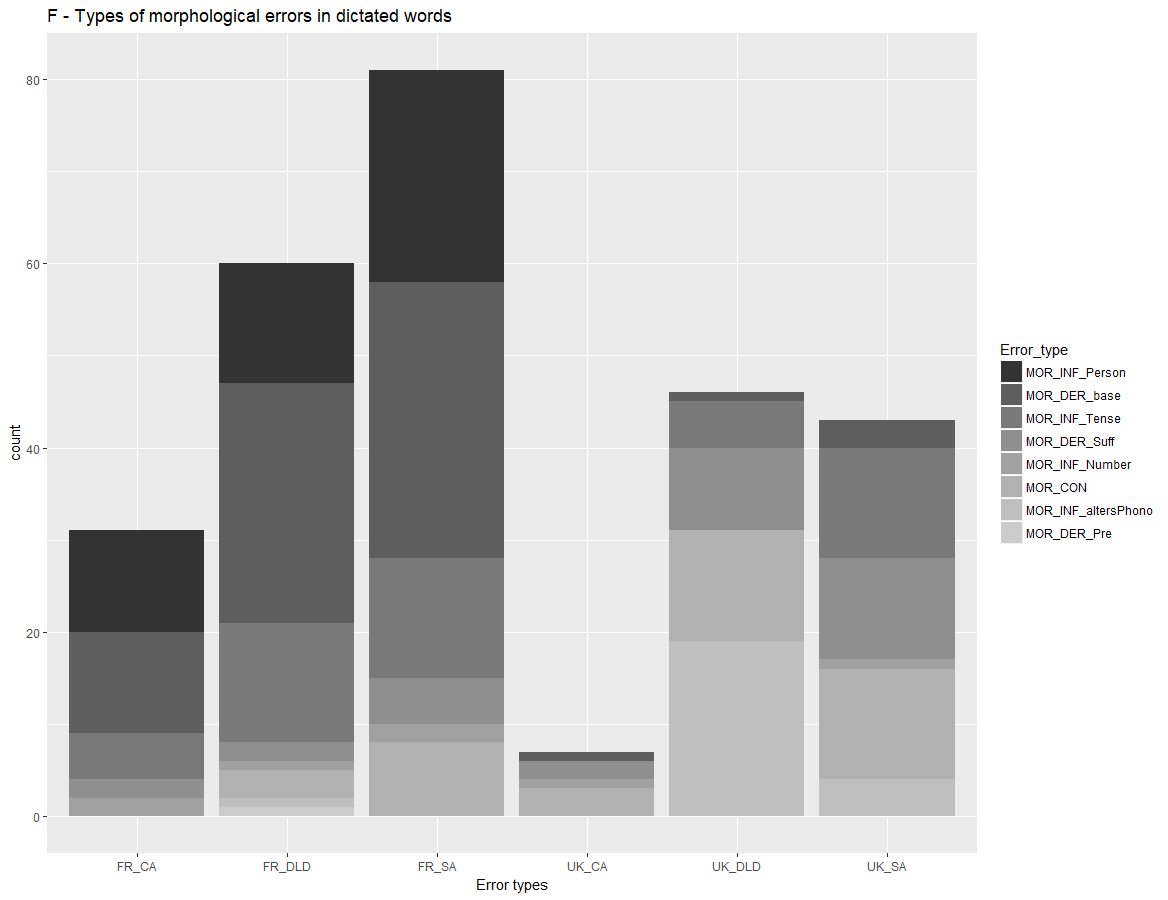


|  | FR_CA | FR_DLD | FR_SA | UK_CA | UK_DLD | UK_SA |
| --- | --- | --- | --- | --- | --- | --- |
| MOR_INF_Person | 11 | 13 | 23 | 0 | 0 | 0 |
| MOR_DER_base | 11 | 26 | 30 | 1 | 1 | 3 |
| MOR_INF_Tense | 5 | 13 | 13 | 0 | 5 | 12 |
| MOR_DER_Suff | 2 | 2 | 5 | 2 | 9 | 11 |
| MOR_INF_Number | 2 | 1 | 2 | 1 | 0 | 1 |
| MOR_CON | 0 | 3 | 8 | 3 | 12 | 12 |
| MOR_INF_altersPhono | 0 | 1 | 0 | 0 | 19 | 4 |
| MOR_DER_Pre | 0 | 1 | 0 | 0 | 0 | 0 |

*Notes*. MOR-INF- Person: Error on person marking, MOR-DER-base: Error on the base of a complex word, MOR-INF-tense: Error on tense inflection, MOR-DER-Suff: Error on the suffix of a complex word, MOR-INF-Number: Error on number marking, MOR-CON: Errors on word contractions, MOR-INF-altersPhono: Omission of a morphological marker affecting phonology, MOR-DER-Pre: Error on the prefix of a complex word

# Appendix G: Fine-grained count of semantic errors in texts


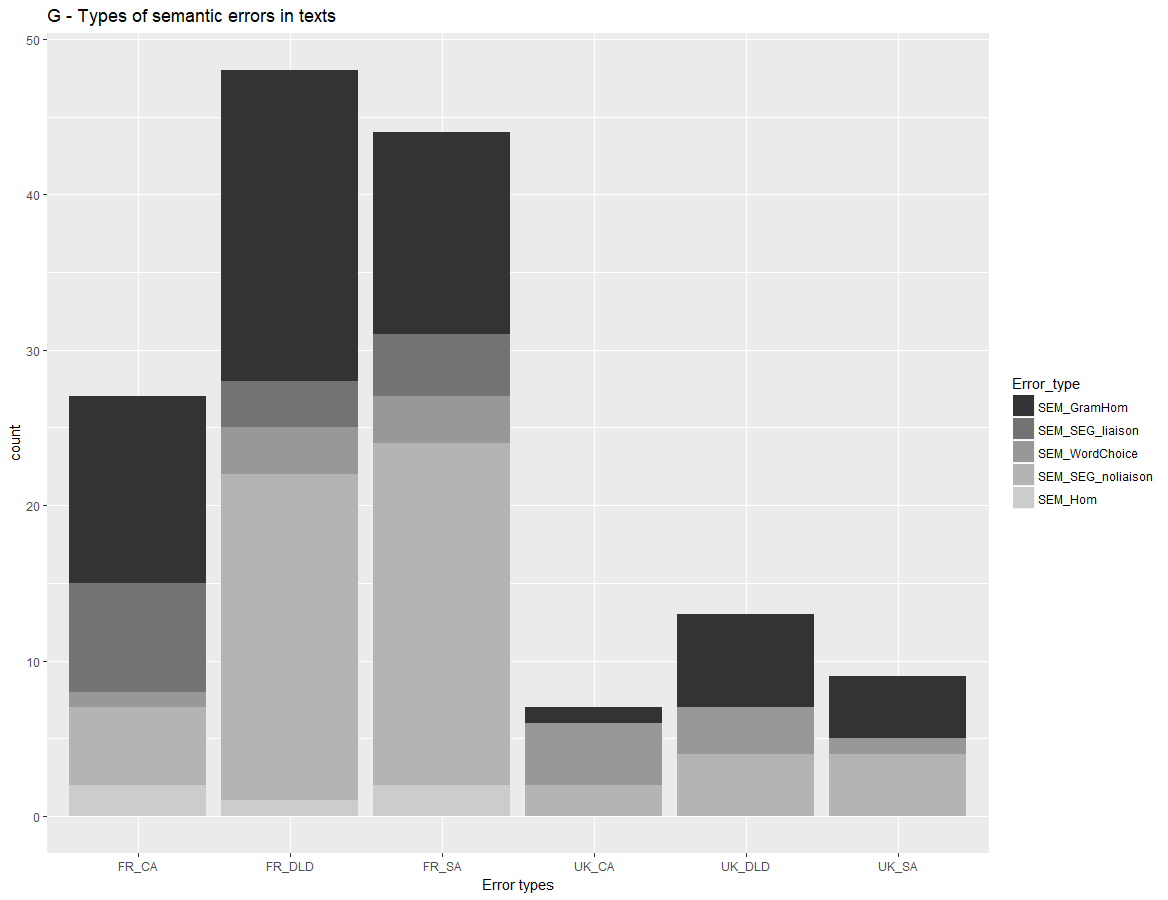


|  | FR_CA | FR_DLD | FR_SA | UK_CA | UK_DLD | UK_SA |
| --- | --- | --- | --- | --- | --- | --- |
| SEM_GramHom | 12 | 20 | 13 | 1 | 6 | 4 |
| SEM_SEG_liaison | 7 | 3 | 4 | 0 | 0 | 0 |
| SEM_WordChoice | 1 | 3 | 3 | 4 | 3 | 1 |
| SEM_SEG_noliaison | 5 | 21 | 22 | 2 | 4 | 4 |
| SEM_Hom | 2 | 1 | 2 | 0 | 0 | 0 |

*Notes*. SEM-GramHom: Use of a grammatical homophone, SEM-SEG-liaison: Segmentation errors (because of a liaison), SEM-WordChoice: Wrong word choice: use of another word, affecting semantics and phonology, SEM-SEG-noliaison: Segmentation errors (without liaison), SEM-HOM: Homophone errors (within the same grammatical category)

# Appendix H: Fine-grained count of semantic errors in 12 dictated words


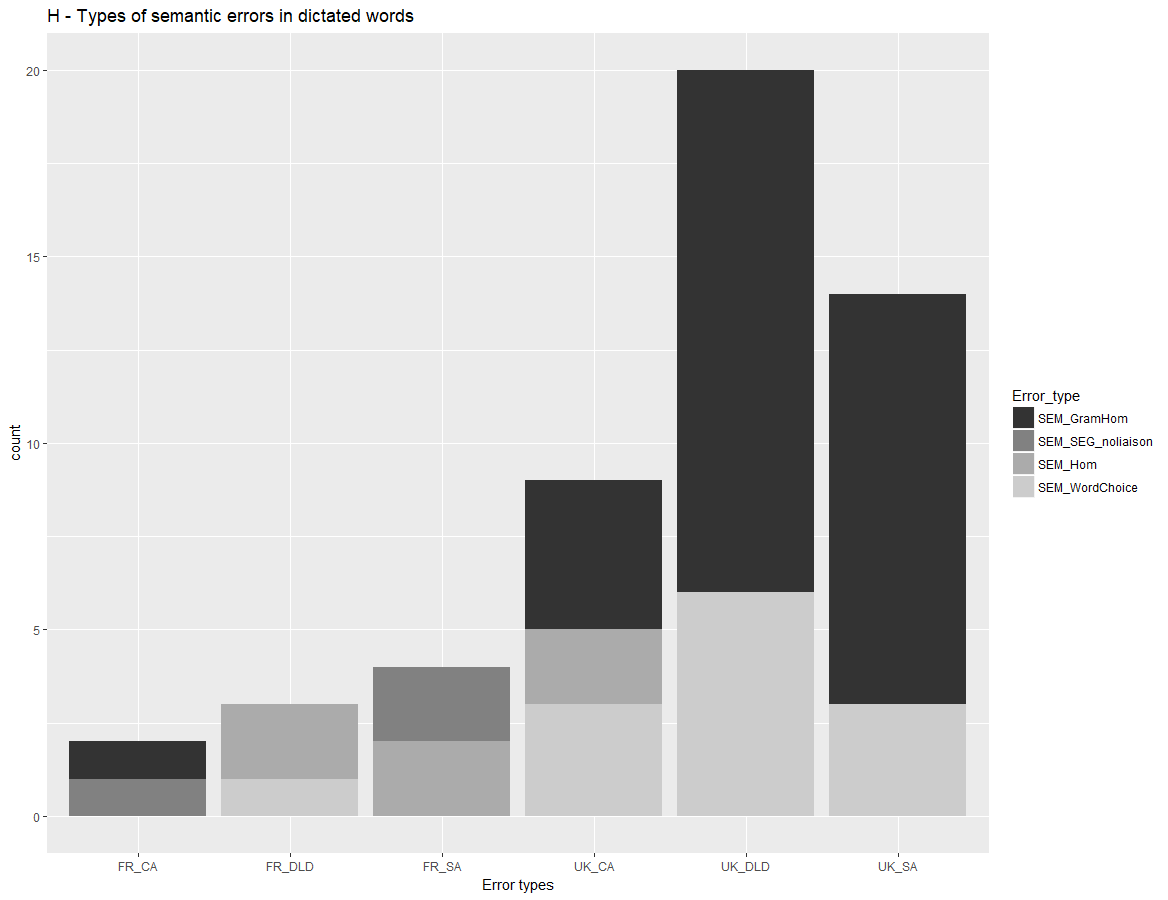


|  | FR_CA | FR_DLD | FR_SA | UK_CA | UK_DLD | UK_SA |
| --- | --- | --- | --- | --- | --- | --- |
| SEM_GramHom | 1 | 0 | 0 | 4 | 14 | 11 |
| SEM_SEG_noliaison | 1 | 0 | 2 | 0 | 0 | 0 |
| SEM_Hom | 0 | 2 | 2 | 2 | 0 | 0 |
| SEM_WordChoice | 0 | 1 | 0 | 3 | 6 | 3 |

*Notes*. SEM-GramHom: Use of a grammatical homophone, SEM-SEG-noliaison: Segmentation errors (without liaison), SEM-HOM: Homophone errors (within the same grammatical category), SEM-WordChoice: Wrong word choice: use of another word, affecting semantics and phonology
